# Supplementary material for: Knowledge, perceptions, and use of psychedelics for mental health among autistic adults: An online survey
Source: PLOS Ment Health. 2025 Dec 26;2(12):e0000514. doi: 10.1371/journal.pmen.0000514 (PMC12798463; doi:10.1371/journal.pmen.0000514)
Supplement: S1 Text — (DOCX) [file pmen.0000514.s001.docx]

**Knowledge, Perceptions, and Use of Psychedelics for Mental Health among Autistic Adults: An Online Survey**

Sahba Afsharnia^1,2^, Vivian Liang^1,3^, Yona Lunsky^1,4^, Aaron P. Orsini^5^, Ami Tint^6^, Hsiang-Yuan Lin^1,2,4*^

**S1 Text: Survey**

**Thank you for participating in our survey! Please answer the following questions as best as you can. You can skip any questions. It may take 20 to 30 minutes to complete.**

**Section 1 - Eligibility and Demographics**

**Eligibility**

1. Please click below to begin or exit the survey.

_Begin survey

_Exit survey

2. Have you completed this survey before?

_Yes

_No

*Branching logic (if yes # 2):*

**Thank you very much for your time to complete this survey.**

*Branching logic (if no # 2):*

**Please type in your email address here to ensure there is no replicate response. Your email address will not be saved for this purpose.**

3. Do you read, write, and speak English fluently?

_Yes

_No

*Branching logic (if no # 3):*

**Thank you very much for your time to complete this survey.**

4. Are you 18 years or older?

_Yes

_No

*Branching logic (if no # 4):*

**Thank you very much for your time to complete this survey.**

5. Have you been diagnosed as Autistic by a mental health professional?

_Yes

_No

*Branching logic (if no # 5): Move on to # 5.1*

5.1 Do you self-identify as Autistic?

_Yes

_No

*Branching logic (if no # 5.1):*

**Thank you very much for your time to complete this survey.**

*Branching logic (if yes either # 5 or # 5.1): Move on to # 6*

**Demographics: We want to learn information about the people who are completing this survey.**

6. What sex were you assigned at birth, on your original birth certificate?

_Male

_Female

_Intersex

_Other: ______________ (please specify)

_I prefer not to answer

7. How do you describe your gender identity? Select all that apply

_Cisgender woman

_Cisgender man

_Nonbinary

_Two-Spirit

_Genderqueer

_Genderfluid

_other (open text)

_I prefer not to answer

8. How old are you?

9. What is the highest level of education you have completed?

_Some high school or less

_ High school diploma or equivalent

_Some college or university

_College or university degree

_Some graduate/professional school

_Graduate/professional degree

_Other: ______________ (please specify)

10. What is your marital status?

_Single

_In a committed relationship (not married)

_Married

_Separated

_Divorced

_Widowed

11. In what country do you currently live?

11.1 Please type in first three digits/characters of your current postal code

11.2 Please leave your email. The information will not be stored and will only be used to prevent replicating responses.

12. Which of the following best describes your racial background?

_White

_South Asian (e.g., East Indian, Pakistani, Sri Lankan, etc.)

_Chinese

_Black

_Filipino

_Latin American

_Arab

_Southeast Asian (e.g., Vietnamese, Cambodian, Laotian, Thai, etc.)

_West Asian (e.g., Iranian, Afghan, etc.)

_Korean

_Japanese

_First Nation

_Unknown or not reported

_Other (specify)_________________________________

13. What is your current employment status?

_Full-time

_Part-time

_Seeking opportunities currently

_Retired

_Prefer not to say

_Other (specify)_________________________________

14. Have you struggled with anxiety, depression, substance misuse, or another psychiatric disorder (e.g., schizophrenia) at any point in your life?

_Yes

_No

*Branching logic (If yes # 14): Move on to # 15*

*Branching logic (If no # 14): Move on to # 16*

15. Please mark all mental health conditions that you have experienced.

_Anxiety Disorder (Social Anxiety, Generalized Anxiety, Panic, Phobia, etc.)

_Eating Disorder (Anorexia, Bulimia, etc.)

_Impulse Control Disorder (Pyromania, Compulsive Gambling, etc.)

_Mood Disorder (Depression, Mania, Bipolar, etc.)

_Post-traumatic Stress Disorder (PTSD)

_Obsessive Compulsive Disorder (OCD)

_Personality Disorder (Paranoid, Avoidant, Borderline, Narcissistic, etc.)

_Psychotic Disorder (Schizophrenia, Schizoaffective, etc.)

_Substance­ Related Disorder (Alcohol or Drug Dependence)

_Neurodevelopmental Disorders (Attention-deficit/hyperactivity disorder, ADHD, Learning Disorders, Tics, etc.

_Burnout, Inertia, Meltdown, and Shutdown

_Existential Crisis

_Other (please specify)

16. Do you use any of the following medications or drugs regularly (at least once a week)? Select all that apply, regardless whether they are prescribed or not.

_Alcohol

_“bath salt” drug products (MDPV, mephedrone, methylone, etc.)

_Benzodiazepines (Xanax, Valium, Klonopin, etc.)

_Caffeine (coffee, tea, soda, dark chocolate, Vivarin, No­Doz, etc.)

_Cannabis (marijuana)

_Cocaine

_Dextromethorphan (DXM)

_Hallucinogens (psilocybin, LSD, DMT, Ayahuasca, mescaline, etc.)

_Hash oil, dabs, THC oil, cannabis extract oils, wax, etc.

_Ketamine

_MDMA (ecstasy, Molly)

_Methamphetamine

_Opioids (heroin, morphine, hydrocodone, oxycodone, codeine, Oxycontin, Vicodin, Percocet, etc.)

_Prescription antidepressants (Celexa, Cymbalta, Effexor, Prozac, Zoloft, etc.)

_Prescription stimulants (amphetamine, methylphenidate, Adderall, Ritalin, Dexedrine, etc.)

_Synthetic marijuana (K2, Spice, etc.)

_Tobacco (nicotine)

_I do not use any of these medications or drugs regularly.

_Other (specify)_________________________________

**Section 2: Interest in, knowledge, and perceptions of psychedelics**

**Now we will ask you questions about your interest in, knowledge of, and perceptions of psychedelics. When we mention psychedelics, we mean: psilocybin (magic) mushrooms, LSD, morning glory seeds, mescaline, peyote cactus, San Pedro cactus, DMT, Ayahuasca, or MDMA (ecstasy, Molly)**

17. How much do you think you know about psychedelics [psilocybin (magic) mushrooms, LSD, morning glory seeds, mescaline, peyote cactus, San Pedro cactus, DMT, Ayahuasca, or MDMA (ecstasy, Molly)]?

_I don’t know a lot

_Slightly knowledgeable

_Moderately knowledgeable

_Very knowledgeable

_Extremely knowledgeable

18. Are psychedelics decriminalized where you live?

_Yes

_No

_I don’t know

19. To the best of your knowledge, are psychedelics being researched for therapeutic potential?

_Yes

_No

20. How dangerous do you think psychedelics are in terms of their risk to the user’s health?

_Not at all dangerous

_Slightly dangerous

_Moderately dangerous

_Very dangerous

_Extremely dangerous

_I don’t know

_Other (specify)_________________________________

21. How interested are you in learning more about psychedelics and how they can be used as part of medical or psychiatric treatment?

_Not at all interested

_Slightly interested

_Moderately interested

_Very interested

_Extremely interested

22. How helpful do you think psychedelics could be in treating mental health conditions commonly experienced by Autistic adults (e.g., Depression, Anxiety, PTSD, etc)?

_Not helpful

_Somewhat helpful

_Moderately helpful

_Very helpful

_Extremely helpful

_I don't know

23. If the opportunity existed, how likely would you be to participate in a government (like Health Canada or FDA)-approved research study examining the use of psychedelics for treating your mental health concerns?

_Not at all likely

_Slightly likely

_Moderately likely

_Very likely

_Extremely likely

_I don’t know

24. Which of the following reasons might prevent you from participating in a clinical trial of psychedelics? (Check all that apply)

_The legal status of psychedelics

_The health risks of psychedelics

_Past knowledge/experience with psychedelics

_Financial, work, or transportation-related limitations

_Religious Beliefs

_Spiritual beliefs

_My mental health concerns are adequately managed with other treatments

_I don’t think psychedelics would help with my concerns

_Other (specify)_________________________________

_None of these would prevent me from participating in a trial

25. Have you ever taken psychedelics? For the purposes of this survey, please count only the following substances: psilocybin (magic) mushrooms, LSD, morning glory seeds, mescaline, peyote cactus, San Pedro cactus, DMT, Ayahuasca, or MDMA (ecstasy, Molly).

_Yes

_No

_I prefer not to say

*Branching logic (if yes # 25): Move on to Section 3*

*Branching logic (if no # 25): Move on to # 26*

26. Are you willing to try psychedelics in the future?

_Yes

_No

_Unsure

*Branching logic (if no # 26):*

**Thank you very much for your time to complete this survey.**

*Branching logic (if yes # 26):*

27. If we are conducting a clinical trial of psychedelics in the future, what would make you feel safe?

**Thank you very much for your time to complete this survey.**

**Section 3: Experience with psychedelics**

**Now we would like to learn more about your past experiences with psychedelics**

28. How old were you when you first took a psychedelic substance? For the purposes of this survey, please count only the following substances: psilocybin (magic) mushrooms, LSD, morning glory seeds, mescaline, peyote cactus, San Pedro cactus, DMT, Ayahuasca, or MDMA (ecstasy, Molly).

29. When did you last use a psychedelic substance? For the purposes of this survey, please count only the following substances: psilocybin (magic) mushrooms, LSD, morning glory seeds, mescaline, peyote cactus, San Pedro cactus, DMT, Ayahuasca, or MDMA (ecstasy, Molly).

_In the past 24 hours

_In the past week

_In the past month

_In the past year

_In the past 5 years

_In the past 10 years

_More than 10 years ago

_I don’t know

30. On approximately how many separate occasions have you taken each of these substances during your lifetime?

|  | # of uses |
| --- | --- |
| Psilocybin (magic) mushrooms | _Never  _1-9 occasions  _10 occasions |
| LSD | _Never  _1-9 occasions  _10 occasions |
| Morning glory seeds | _Never  _1-9 occasions  _10 occasions |
| Mescaline | _Never  _1-9 occasions  _10 occasions |
| Peyote cactus | _Never  _1-9 occasions  _10 occasions |
| San Pedro cactus | _Never  _1-9 occasions  _10 occasions |
| DMT | _Never  _1-9 occasions  _10 occasions |
| Ayahuasca | _Never  _1-9 occasions  _10 occasions |
| MDMA (ecstasy, Molly) | _Never  _1-9 occasions  _10 occasions |

31. Approximately how many times have you taken each of these substances over the past year?

|  | # of uses |
| --- | --- |
| Psilocybin (magic) mushrooms | _Never  _1-9 occasions  _10 occasions |
| LSD | _Never  _1-9 occasions  _10 occasions |
| Morning glory seeds | _Never  _1-9 occasions  _10 occasions |
| Mescaline | _Never  _1-9 occasions  _10 occasions |
| Peyote cactus | _Never  _1-9 occasions  _10 occasions |
| San Pedro cactus | _Never  _1-9 occasions  _10 occasions |
| DMT | _Never  _1-9 occasions  _10 occasions |
| Ayahuasca | _Never  _1-9 occasions  _10 occasions |
| MDMA (ecstasy, Molly) | _Never  _1-9 occasions  _10 occasions |

32. What sort of dosing do you typically use?

_Small doses/microdoses only (sub-hallucinogenic/sub-perceptual doses)

_Mixed use of various doses, from small to full doses

_Full doses only (hallucinogenics)

33. What was your intention for taking a psychedelic? (Check all that apply):

_I took a psychedelic only because other people were, but I did not have a serious intention.

_Curiosity without any other serious intention.

_Recreational (e.g., to enjoy the experience, which may or may not involve a social recreational event such as a  concert).

_A serious intention for psychological self­ exploration (e.g., resolve a personal issue or to increase self-understanding).

_A serious intention to explore spirituality or the sacred (e.g., to contemplate God, as you understand that word, or the nature of ultimate reality, and so on).

_A premeditated intention to help with mental health concerns.

_Other (specify)_________________________________

34. Did your past psychedelic use help with your mental health concerns?

_Yes

_No

_I don’t know / Not sure

_Other (specify)_________________________________

*Branching logic (if yes or other or I don’t know / Not sure  # 34): Move on to # 35*

*Branching logic (if no # 34): Move on to # 48*

35. Which of these mental health conditions improved after using psychedelics  (Check all that apply):

_Anxiety Disorder (Social Anxiety, Generalized Anxiety, Panic, Phobia, etc.)

_Eating Disorder (Anorexia, Bulimia, etc.)

_Impulse Control Disorder (Pyromania, Compulsive Gambling, etc.)

_Mood Disorder (Depression, Mania, Bipolar, etc.)

_Post-traumatic Stress Disorder (PTSD)

_Obsessive Compulsive Disorder (OCD)

_Personality Disorder (Paranoid, Avoidant, Borderline, Narcissistic, etc.)

_Psychotic Disorder (Schizophrenia, Schizoaffective, etc.)

_Substance­ Related Disorder (Alcohol or Drug Dependence)

_Neurodevelopmental Disorders (Attention-deficit/hyperactivity disorder, ADHD, Learning Disorders, Tics, etc.

_Burnout, Inertia, Meltdown, and Shutdown

_Existential Crisis

_Other (please specify)

**For this section we would like you to focus on one psychedelic experience that you feel was particularly helpful for your mental health.**

36. How old were you when the psychedelic experience that improved your mental health took place?

37. How would you describe your mental health improvement after this psychedelic experience?

_Stopped experiencing the mental health concerns completely since the experience (full remission).

_Greatly reduced experiencing the mental health concern(s) since the experience.

_Reduced experiencing the mental health concern(s) somewhat since the experience.

_Initially stopped experiencing the mental health concern(s) **completely**, then the mental health concern(s) returned to the same level as before.

_Stopped experiencing the mental health concern(s) somewhat for a period of time, then the mental health concern(s) returned at the same level as before.

_Other (please specify)

38. How long did your mental health improvement last?

_Less than 1 week

_ 1 -­ 2 weeks

_ 3 -­ 4 weeks

_ 1 -­ 3 months

_ 4 ­- 6 months

_ 7 -­ 12 months

_ 1 -­ 2 years

_ 3 -­ 5 years

_ 6 -­ 10 years

_ 11 -­ 20 years

_ More than 20 years

39. Which substance led to the psychedelic experience associated with your improvement in mental health concerns?

_ psilocybin mushrooms

_LSD

_morning glory seeds

_mescaline (pure compound)

_peyote cactus

_San Pedro cactus

_DMT (pure compound)

_Ayahuasca

_MDMA (ecstasy, Molly)

_Other (specify)_________________________________

 40. To the best of your knowledge, what was the approximate dose that led to your improvement in mental health concerns?

_Very low dose/microdoses

_Low dose

_Moderate dose

_High dose

_Very high dose

_Mixed use of various doses, from small to full doses

_I don’t know / Unsure

_Please specify if you remember the exact dose

41. Where did this experience take place? (Check all that apply):

_At home

_At a party

_In a public place (e.g., shopping mall, movie theater, etc.)

_At a concert or festival

_Outdoors in nature

_In a religious or spiritual setting (e.g., ceremony, retreat, etc.)

_Other (please specify)

**Thinking about the overall psychedelic experience that led to improvements in your mental health, please rate the degree to which you experienced the following phenomena. Answer each question according to your feelings, thoughts, and experiences at that time**

42. How personally meaningful was the psychedelic experience that led to mental health improvement, and your contemplation of that experience?

_0 No more than routine, everyday experiences (not meaningful at all)

_1 Similar to meaningful experiences that occur on average once or more a week (slightly meaningful)

_2 Similar to meaningful experiences that occur on average once a month (mildly meaningful)

_3 Similar to meaningful experiences that occur on average once a year (moderately meaningful)

_4 Among the 5 most meaningful experiences of my life (strongly meaningful)

_5 The single most meaningful experience of my life (extremely meaningful: more than any other meaningful experiences in my life and stronger than 4)

43. Indicate the degree to which the psychedelic experience that led to your improvement in mental health issues, and your contemplation of that experience, were spiritually significant to you.

_0 Not at all

_1 Slightly

_2 Moderately

_3 Very much

_4 Among the 5 most spiritually significant experiences of my life

_5 The single most spiritually significant experience of my life

44. How psychologically challenging was the psychedelic experience that led to improvements in your mental health concerns?

_0 No more than routine, everyday experiences (not challenging at all)

_1 Similar to difficult or challenging experiences that occur on average once or more a week (slightly challenging)

_2 Similar to difficult or challenging experiences that occur on average once a month (mildly challenging)

_3 Similar to difficult or challenging experiences that occur on average once a year (moderately challenging)

_4 Among the 5 most difficult or challenging experiences of my life (strongly challenging)

_5 The single most difficult or challenging experience of my life (extremely challenging: more than any other challenging experiences in my life and stronger than 4)

45. How psychologically insightful to you was the psychedelic experience that led to your improvement in mental health concerns, and your contemplation of that experience?

_0 No more than routine, everyday psychologically insightful experiences (not psychologically insightful at all)

_1 Similar to psychologically insightful experiences that occur on average once or more a week (slightly psychologically insightful)

_2 Similar to psychologically insightful experiences that occur on average once a month (mildly psychologically insightful)

_3 Similar to psychologically insightful experiences that occur on average once a year (moderately psychologically insightful)

_4 Among the 5 most psychologically insightful experiences of my life (strongly psychologically insightful)

_5 The single most psychologically insightful experience of my life (extremely psychologically insightful: more than any other psychologically insightful experiences in my life and stronger than 4)

46. Please select items that were related to your psychedelic­ associated improvement in mental health concerns. Check all that apply.

_Strengthening your belief in your own ability to recover.

_Reducing stress involved with recovering.

_Reframing mental health improvement as a spiritual task.

_Changing life priorities or values.

_Changing your orientation toward the future, so that long­term benefits outweighed immediate desires/preoccupations.

47. Please describe any additional reasons (not listed above) how your psychedelic experience contributed to your mental health improvement.

48. Did you experience any other behavioral changes after this psychedelic experience? Check all that apply, and please provide any relevant details in the Comments box below.

_None

_Reduced or stopped using other drugs

_Started using other drugs more often / heavily

_Changes in diet / nutrition

_Increased physical activity / exercise

_Decreased physical activity / exercise

_Improved relationships with others

_Worsened relationships with others

_Improvements in career / work life

_Worsening of career / work life

_Other / comments: ____

49. Did you experience any persisting negative effects from this psychedelic experience? By persisting, we mean negative effects that lasted beyond the acute (immediate) period of drug effects.

_Yes.

_No.

_Not sure.

50. Please describe any negative or potentially negative persisting effects you may have experienced as a result of this psychedelic session.

51. Overall, how would you rate the severity of these negative effects?

_Not at all severe

_Slightly severe

_Moderately severe

_Very severe

_Extremely severe

52. Is there anything else you would like us to know about your psychedelic experiences?

**Thank you very much for your time and participation. You have completed the survey, providing us with critical information about your knowledge and perception surrounding psychedelics, and/or your experience of improvement in mental health concerns after a psychedelic experience.**

**Please feel free to share this survey information with any other interested parties you may know.**

**If you are interested in sharing more of your psychedelic experience in a researcher-conducted qualitative interview in the future (another independent project), please leave your email here ____. This email will be stored separately from your survey data and we will not be able to connect your email to your survey answers. We will contact you regarding participation in other qualitative studies in the future.**

**Have a nice day!**
