# Supplementary material for: Knowledge, perceptions, and use of psychedelics for mental health among autistic adults: An online survey
Source: PLOS Ment Health. 2025 Dec 26;2(12):e0000514. doi: 10.1371/journal.pmen.0000514 (PMC12798463; doi:10.1371/journal.pmen.0000514)
Supplement: S1 Table — (DOCX) [file pmen.0000514.s002.docx]

**Knowledge, Perceptions, and Use of Psychedelics for Mental Health among Autistic Adults: An Online Survey**

Sahba Afsharnia^1,2^, Vivian Liang^1,3^, Yona Lunsky^1,4^, Aaron P. Orsini^5^, Ami Tint^6^, Hsiang-Yuan Lin^1,2,4*^

**Supporting Information File: S1 Table**

S1 Table: Knowledge and perception, stratified by sex, education, age, marital status, and country of residence.

**S1 Table: Knowledge and perception, stratified by sex, education, age, marital status, and country of residence**

|  | **Whole Sample**  **(N=257)** | **Sex** | | | **Education** | | **Age** | | **Marital Status** | | **Country of Residence** | |
| --- | --- | --- | --- | --- | --- | --- | --- | --- | --- | --- | --- | --- |
|  |  | **Assigned female at birth (n=162)** | **Assigned male at birth (n=91)** | **Other (n=4)** | **Completion of Secondary Education or less (n=83)** | **Completion of Post-Secondary Education (n=174)** | **Age <40 (n=162)** | **Age ≥40 (n=95)** | **Single (n=130)** | **Married / Partnered (n=127)** | **Canadian (n=115)** | **Non- Canadian (n=142)** |
| **Perceived Knowledge of Psychedelics** | | | | | | | | | | | | |
| Low (“I don’t know” or “slightly”) | 40.1% (103/257) | 45.7% (74/162) | 30.8% (28/91) | 25.0%  (1/4) | 45.8%  (38/83) | 37.9%  (66/174) | 43.8% (71/162)^4^ | 33.7% (32/95) | 49.9%  (61/130) | 33.1% (42/127) | 55.7% (64/115)^4^ | 27.5%  (39/142) |
| Moderate | 32.7% (84/257) | 32.7% (53/162) | 33.0% (30/91) | 25.0% (1/4) | 31.3%  (26/83) | 33.3%  (58/174) | 26.5% (43/162)^4^ | 43.2% (41/95) | 30.8%  (40/130) | 34.6% (44/127) | 22.6% (26/115)^4^ | 40.8%  (58/142) |
| High (“very” or “extremely”) | 27.2% (70/257) | 21.6% (35/162) | 36.3% (33/91) | 50.0%  (2/4) | 22.9%  (19/83) | 28.7%  (50/174) | 29.6% (48/162)^4^ | 23.2% (22/95) | 22.3% (29/130) | 32.3% (41/127) | 21.7% (25/115)^4^ | 31.7%  (45/142) |
| **Are you willing to try psychedelics in the future?** | | | | | | | | | | | | |
| Yes | 77.8% (200/257) | 75.9% (123/162) | 80.2% (73/91) | 100.0% (4/4) | 73.5%  (61/83) | 79.3%  (138/174) | 77.6% (125/162) | 78.9% (75/95) | 76.9% (100/130) | 78.7% (100/127) | 68.7% (79/115)^4^ | 85.2% (121/142) |
| Maybe | 14.4% (37/257) | 16.0% (26/162) | 12.1% (11/91) | 0.0%  (0/4) | 13.3%  (11/83) | 14.9%  (26/174) | 13.0% (21/162) | 16.8% (16/95) | 13.8% (18/130) | 15.0% (19/127) | 21.7% (25/115)^4^ | 8.5%  (12/142) |
| No | 7.8% (20/257) | 8.0% (13/162) | 7.7% (7/91) | 0.0%  (0/4) | 13.3%  (11/83) | 5.2%  (9/174) | 9.9% (16/162) | 4.2% (4/95) | 9.2% (12/130) | 6.3% (8/127) | 9.6 (11/115)^4^ | 6.3%  (9/142) |
| **Likeliness to Participate in a Government Trial**^2^ | | | | | | | | | | | | |
| Low (“not at all likely” or “slightly likely”) | 14.8% (38/256) | 17.4% (28/161) | 11.0% (10/91) | 0.0%  (0/4) | 18.1%  (15/83) | 13.3%  (23/173) | 16.8% (27/161) | 11.6% (11/95) | 13.8% (18/130) | 15.7% (20/127) | 15.8% (18/114) | 14.1%  (20/142) |
| Moderate | 10.2% (26/256) | 9.9% (16/161) | 9.9% (9/91) | 25.0%  (1/4) | 8.4%  (7/83) | 11.0%  (19/173) | 9.3% (15/161) | 11.6% (11/95) | 6.9% (9/130) | 11.8% (15/127) | 13.2% (15/114) | 7.7%  (11/142) |
| High (“very likely” or “extremely likely”) | 72.7% (186/256) | 71.4% (115/161) | 74.7% (68/91) | 75.0%  (3/4) | 69.9%  (58/83) | 74.0%  (128/173) | 70.8% (114/161) | 75.8% (72/95) | 75.4% (98/130) | 69.3% (88/127) | 67.5% (77/114) | 76.8% (109/142) |
| I don’t know | 2.3% (6/256) | 1.2% (2/161) | 4.4% (4/91) | 0.0%  (0/4) | 3.6%  (3/83) | 1.7%  (3/173) | 3.1% (5/161) | 1.0% (1/95) | 2.3% (3/130) | 2.4% (3/127) | 3.5% (4/114) | 1.4%  (2/142) |
| **Interest in Learning about Psychedelics** | | | | | | | | | | | | |
| Low  (“not at all interested” or “slightly interested) | 11.3% (29/257) | 11.1% (18/162) | 12.1% (11/61) | 0.0%  (0/4) | 14.5%  (12/83) | 9.8%  (17/174) | 13.6% (22/162) | 7.4% (7/95) | 14.6% (19/130) | 7,8% (10/127) | 15.7% (18/115)^4^ | 7.7%  (11/142) |
| Moderate (“moderately interested”) | 12.1% (31/257) | 11.7% (19/162) | 13.1% (12/91) | 0.0%  (0/4) | 12.0%  (10/83) | 12.1%  (21/174) | 11.7% (19/162) | 12.6% (12/95) | 11.5% (15/130) | 12.6% (16/127) | 15.7% (18/115)^4^ | 9.2%  (13/142) |
| High (“very interested” or “extremely interested”) | 76.7% (197/257) | 77.2% (125/162) | 74.7% (68/91) | 100.0% (4/4) | 13.5%  (61/83) | 78.2%  (136/174) | 74.7% (121/162) | 80.0% (76/95) | 73.8% (96/130) | 79.5% (101/127) | 68.7% (79/115)^4^ | 83.1% (118/142) |
| **Perceived Helpfulness of Psychedelics** | | | | | | | | | | | | |
| Low  (“not helpful” or “somewhat”) | 8.2% (21/257) | 9.3% (15/162) | 6.6% (6/91) | 0.0%  (0/4) | 10.8%  (9/83) | 6.9%  (12/174) | 9.3% (15/162) | 6.3% (6/95) | 7.7% (10/130) | 7.1% (9/127) | 13.0% (15/115) | 4.2%  (6/142) |
| Moderate | 9.7% (25/257) | 11.1% (18/162) | 7.7% (7/91) | 0.0%  (0/4) | 9.6%  (8/83) | 9.8%  (17/174) | 11.1% (18/162) | 7.4% (7/95) | 9,2% (12/130) | 10.2% (13/127) | 13.9% (16/115) | 6.3%  (9/142) |
| High (“very” or “extremely helpful”) | 69.7% (179/257) | 67.3% (109/162) | 72.5% (66/91) | 100.0% (4/4) | 68.7%  (57/83) | 70.1%  (122/174) | 70.4% (114/162) | 68.4% (65/95) | 70.8% (92/130) | 68.5% (87/127) | 53.9% (62/115) | 82.4% (117/142) |
| I don’t know | 12.1% (31/257) | 12.3% (20/162) | 13.2% (12/91) | 0.0%  (0/4) | 10.8%  (9/83) | 13.2%  (23/174) | 9.3% (15/162) | 17.9% (17/95) | 12.3% (16/130) | 12.6% (16/127) | 19.1% (22/115) | 7.0%  (10/142) |
| **Are Psychedelics Decriminalized Where You Live?** | | | | | | | | | | | | |
| Yes | 15.2% (39/257) | 13.6% (22/162) | 17.6% (16/91) | 25.0%  (1/4) | 9.6%  (8/83) | 17.8%  (31/174) | 13.6% (22/162) | 17.9% (17/95) | 16.2% (21/130) | 14.2% (18/127) | 7.8% (9/115)^4^ | 21.1%  (30/142) |
| No | 61.5% (158/257) | 60.5% (98/162) | 62.6% (57/91) | 75.0%  (3/4) | 61.4%  (51/83) | 61.5%  (107/174) | 59.3% (96/162) | 65.3% (62/95) | 58.5% (76/130) | 64.6% (82/127) | 53.0% (61/115)^4^ | 68.3%  (97/142) |
| I don’t know | 23.3% (60/257) | 25.9% (42/162) | 19.8% (18/91) | 0.0%  (0/4) | 28.9%  (24/83) | 20.7%  (36/174) | 27.2% (44/162) | 16.8% (16/95) | 25.4% (33/130) | 21.3% (27/127) | 39.1% (45/115)^4^ | 10.7%  (15/142) |
| **To the best of your knowledge, are psychedelics being researched for therapeutic potential?** | | | | | | | | | | | | |
| Yes | 91.4% (234/256) | 92.6% (150/162) | 88.9% (80/90) | 100.0% (4/4) | 91.5%  (75/82) | 8.6%  (15/174) | 90.7% (146/161) | 92.6% (88/95) | 91.5% (119/130) | 91.3% (115/126) | 6.1% (7/114) | 89.4% (127/142) |
| No | 8.6% (22/256) | 7.4% (12/162) | 11.1% (10/90) | 0.0%  (0/4) | 8.5%  (7/82) | 91.4%  (159/174) | 9.3% (15/161) | 7.4% (7/95) | 8.5% (11/130) | 8.7% (11/126) | 93.9% (107/114) | 10.6%  (15/142) |
| **What Might prevent you from trying Psychedelics?**^3^ | | | | | | | | | | | | |
| Legal status of psychedelics | 23.3% (60/257) | 25.3% (41/162) | 19.8% (18/91) | 25.0%  (1/4) | 20.5%  (17/83) | 24.7%  (43/174) | 26.5% (43/162) | 17.9% (17/95) | 20.8% (27/130) | 26.0% (33/127) | 17.4% (20/115) | 28.2%  (40/142) |
| The health risks of psychedelics | 18.3% (47/257) | 19.14% (31/162) | 17.6% (16/91) | 0.0%  (0/4) | 19.3%  (16/83) | 17.8%  (31/174) | 19.8% (32/162) | 15.8% (15/95) | 16.2% (21/130) | 20.5% (26/127) | 23.5% (27/115) | 14.1%  (20/142) |
| Past knowledge/experience with psychedelics | 10.9% (28/257) | 14.8% (24/162) | 4.4% (4/91) | 0.0%  (0/4) | 6.0%  (5/83) | 13.2%  (23/174) | 11.1% (18/162) | 10.5% (10/95) | 10.8% (14/130) | 11.0% (14/127) | 11.3% (13/115) | 10.6%  (15/142) |
| Work or transportation-related limitations | 28.0% (72/257) | 32.1% (52/162) | 19.8% (18/91) | 50.0%  (2/4) | 25.3%  (21/83) | 29.3%  (51/174) | 28.4% (46/162) | 27.4% (26/95) | 30.8% (40/130) | 25.1% (32/127) | 30.4% (35/115) | 26.1%  (37/142) |
| Religious beliefs | 1.6% (4/257) | 1.2% (2/162) | 2.2% (2/91) | 0.0%  (0/4) | 0.0%  (0/83) | 2.3%  (4/174) | 1.9% (3/162) | 1.1% (1/95) | 1.5% (2/130) | 1.6% (2/127) | 1.7% (2/115) | 1.4%  (2/142) |
| Spiritual beliefs | 1.2% (3/257) | 0.6% (1/162) | 2.2% (2/91) | 0.0%  (0/4) | 2.4%  (2/83) | 0.57%  (1/174) | 1.9% (3/162) | 0.0% (0/95) | 2.3% (3/130) | 0.0% (0/127) | 0.9% (1/115) | 1.4%  (2/142) |
| My mental health concerns are adequately managed with other treatments | 13.6% (35/257) | 12.3% (20/162) | 15.4% (14/91) | 25.0%  (1/4) | 15.7%  (13/83) | 12.6%  (22/174) | 15.4% (25/162) | 10.5% (10/95) | 12.1%  (17/130) | 14.2% (18/127) | 17.4% (20/115) | 10.6%  (15/142) |
| I don’t think psychedelics would help with my concerns | 5.4% (14/257) | 5.6% (9/162) | 5.5% (5/91) | 0.0%  (0/4) | 7.2%  (6/83) | 4.6%  (8/174) | 6.8% (11/162) | 3.2% (3/95) | 6.2% (8/130) | 4.7% (6/127) | 8.7% (10/115) | 2.8%  (4/142) |
| Other | 13.6% (35/257) | 12.7% (27/162) | 8.8% (8/91) | 0.0%  (0/4) | 13.3%  (11/83) | 13.8%  (24/174) | 16.0% (26/162) | 9.5% (9/95) | 16.9% (22/130) | 10.2% (13/127) | 13.0% (15/115) | 14.1%  (20/142) |
| None of these reasons would prevent me from participating in a trial | 33.1% (85/257) | 28.4% (46/162) | 41.8% (38/91) | 25.0%  (1/4) | 36.1%  (30/83) | 31.6%  (55/174) | 27.2% (44/162) | 43.2% (41/95) | 32.3% (42/130) | 33.9% (43/127) | 30.4% (35/115) | 35.2%  (50/142) |

^1^This was a multi-select question.

^2^1 out of 257 participants did not respond to these items.

^3^This was a multi-select question.

^4^Significant Chi-square tests (uncorrected p < .05), suggesting significant effects of the identified demographic factors on results.
