# Supplementary material for: Knowledge, perceptions, and use of psychedelics for mental health among autistic adults: An online survey
Source: PLOS Ment Health. 2025 Dec 26;2(12):e0000514. doi: 10.1371/journal.pmen.0000514 (PMC12798463; doi:10.1371/journal.pmen.0000514)
Supplement: S2 Table — (DOCX) [file pmen.0000514.s003.docx]

**Knowledge, Perceptions, and Use of Psychedelics for Mental Health among Autistic Adults: An Online Survey**

Sahba Afsharnia^1,2^, Vivian Liang^1,3^, Yona Lunsky^1,4^, Aaron P. Orsini^5^, Ami Tint^6^, Hsiang-Yuan Lin^1,2,4*^

**Supporting Information File: S2 Table**

S2 Table: General psychedelic experiences, stratified by sex, education, age, marital status, and country of residence

**S2 Table: General psychedelic experiences, stratified by sex, education, age, marital status, and country of residence**

|  | **Whole Sample**  **(N=181)** | **Sex** | | | **Education** | | **Age** | | **Marital Status** | | **Country of Residence** | |
| --- | --- | --- | --- | --- | --- | --- | --- | --- | --- | --- | --- | --- |
|  |  | **Assigned female at birth** | **Assigned male at birth** | **Other** | **Completion of Secondary School Education or less** | **Completion of Post-Secondary School Education** | **Age <40** | **Age ≥40** | **Single** | **Married/ Partnered** | **Canadian** | **Non- Canadian** |
| **Mean age of first psychedelic experience** | 24.7  SD = 10.648 | 23.5  SD=9.265 | 27.0  SD=12.543 | 19.3  SD=1.5 | 21.6  SD=9.677 | 25.7  SD=10.988 | 21.9  SD =  5.163 | 29.4  SD =  15.010 | 25.9  SD =  10.892 | 23.1  SD =  10.293 | 24.4  SD =  11.027 | 24.9  SD = 10.490 |
| **What substance(s) have you tried?^1^** | | | | | | | | | | | | |
| Psilocybin | 91.7% (166/181) | 89.9% (98/109) | 65.3% (64/98) | 100.0% (4/4) | 86.7%  (52/60) | 94.2%  (114/121) | 90.4% (103/114) | 94.0% (63/67) | 91.0% (78/86) | 92.6% (88/95) | 95.2% (60/63) | 89.8% (106/118) |
| LSD | 55.2% (100/181) | 50.5% (55/109) | 42.9% (42/98) | 75.0%  (3/4) | 56.7%  (34/60) | 54.5%  (66/121) | 53.5% (61/114) | 58.2% (39/67) | 52.3% (45/86) | 57.9% (55/95) | 42.9% (27/63) | 61.9% (73/118) |
| Morning glory seeds | 8.8%  (16/181) | 4.6%  (5/109) | 11.2% (11/98) | 0.0%  (0/4) | 3.3%  (2/60) | 11.6%  (14/121) | 7.9%  (9/114) | 7.2%  (7/97) | 12.8% (11/86) | 5.3%  (5/95) | 6.3%  (4/63) | 10.2% (12/118) |
| Mescaline | 8.8%  (16/181) | 7.3%  (8/109) | 7.1%  (7/98) | 25.0%  (1/4) | 6.7%  (4/60) | 9.9%  (12/121) | 6.1%  (7/114) | 9.3%  (9/97) | 8.1%  (7/86) | 9.5%  (9/95) | 9.5%  (6/63) | 8.5% (10/118) |
| Peyote Cactus | 5.5%  (10/181) | 3.7%  (4/109) | 5.1%  (5/98) | 25.0%  (1/4) | 1.7%  (1/60) | 7.4%  (9/121) | 5.3%  (6/114) | 4.1%  (4/97) | 7.0%  (6/86) | 4.2%  (4/95) | 1.6%  (1/63) | 7.6%  (9/118) |
| San Pedro Cactus | 9.4%  (17/181) | 6.4%  (7/109) | 10.2% (10/98) | 0.0%  (0/4) | 5.0%  (3/60) | 11.6%  (14/121) | 11.4% (13/114) | 4.1%  (4/97) | 10.5%  (9/86) | 8.4%  (8/95) | 4.8%  (3/63) | 11.9% (14/118) |
| DMT | 19.3% (35/181) | 11.9% (13/109) | 22.4% (22/98) | 0.0%  (0/4) | 13.3%  (8/60) | 22.3%  (27/121) | 21.9% (25/114) | 10.3% (10/97) | 18.6% (16/86) | 20.0% (19/95) | 9.5%  (4/63) | 26.3% (31/118) |
| Ayahuasca | 13.3% (24/181) | 10.1% (11/109) | 13.3% (13/98) | 0.0%  (0/4) | 8.3%  (5/60) | 15.7%  (19/121) | 12.3% (14/114) | 10.3% (10/97) | 15.1% (13/86) | 11.6% (11/95) | 3.2%  (2/62) | 18.7% (22/118) |
| MDMA | 54.1% (98/181) | 52.3% (57/109) | 40.8% (40/98) | 25.0%  (1/4) | 45.0%  (27/60) | 58.7%  (71/121) | 52.6% (60/114) | 39.1% (38/97) | 53.5% (46/86) | 54.7% (52/95) | 41.3% (26/62) | 61.0% (72/118) |
| Percentage that has only tried 1 substance | 26.5% (48/181) | 31.2% (34/109) | 13.7% (13/98) | 25.0%  (1/4) | 26.7%  (16/60) | 26.4%  (32/121) | 28.1% (32/114) | 16.5% (16/97) | 27.9% (24/86) | 25.3% (24/95) | 38.7% (24/62)^2^ | 20.3% (24/118) |
| Percentage that has tried 2-3 substances | 19.3% (35/181) | 47.7% (52/109) | 28.6% (28/98) | 50.0%  (2/4) | 56.7%  (34/60) | 41.3%  (50/121) | 44.7% (51/114) | 32.0% (31/97) | 39.5% (34/86) | 50.5% (48/95) | 43.5% (27/62)^2^ | 46.6% (55/118) |
| Percentage that has tried 4+ substances | 26.0% (47/181) | 18.3% (20/109) | 26.5% (26/98) | 25.0%  (1/4) | 13.3%  (8/60) | 32.2%  (39/121) | 24.6% (28/114) | 19.6% (19/97) | 29.1% (25/86) | 23.2% (22/95) | 14.5% (9/62)^2^ | 32.2% (38/118) |
| **What sort of dosing do you typically use?**^3^ | | | | | | | | | | | | |
| Small doses/microdoses only | 18.6% (32/172) | 18.1% (19/105) | 20.6% (13/63) | 0.0%  (0/4) | 12.3%  (7/57) | 21.7%  (25/115) | 16.2% (18/111) | 23.0% (14/61) | 17.1% (14/82) | 20.0% (18/90) | 26.2% (16/61) | 14.4% (16/111) |
| Mixed use of various doses, from small to full doses | 60.5% (104/172) | 61.0% (64/105) | 58.7% (37/63) | 75.0%  (3/4) | 64.9%  (37/57) | 58.3%  (67/115) | 61.3% (68/111) | 59.0% (36/61) | 57.3% (47/82) | 63.3% (57/90) | 54.1% (33/61) | 64.0% (71/111) |
| Full doses only | 20.9% (36/172) | 21.0% (22/105) | 20.6% (13/63) | 25.0%  (1/4) | 22.8%  (13/57) | 20.0%  (23/115) | 23.4% (26/111) | 18.0% (11/61) | 25.6% (21/82) | 16.7% (15/90) | 19.7% (12/61) | 21.6% (24/111) |
| **Intention for psychedelic experience^1^** | | | | | | | | | | | | |
| No serious intention, other people were using | 10.0% (18/181) | 11.4% (12/109) | 7.4%  (5/68) | 25.0%  (1/4) | 10.0%  (6/60) | 9.9%  (12/121) | 10.5% (12/114) | 9.0%  (6/67) | 10.5% (9/86) | 9.5%  (9/95) | 15.9% (10/63) | 6.8%  (8/118) |
| Curiosity | 28.2% (51/181) | 29.5% (31/109) | 27.9% (19/68) | 25.0%  (1/4) | 33.3%  (20/60) | 25.6%  (31/121) | 31.6% (36/114) | 22.3% (15/67) | 23.3% (20/86) | 32.6% (31/95) | 34.9% (22/63) | 24.6% (29/118) |
| Recreation | 57.5% (104/181) | 60.0% (63/109) | 55.9% (38/68) | 75.0%  (3/4) | 60.0%  (36/60) | 56.2%  (68/121) | 64.8% (74/114) | 44.8% (30/67) | 54.7% (47/86) | 60.0% (57/95) | 63.5% (40/63) | 54.2% (64/118) |
| Psychological self-exploration | 56.1% (107/181) | 60.0% (63/109) | 58.8% (40/68) | 100.0% (4/4) | 53.3%  (32/60) | 62.0%  (75/121) | 60.5% (69/114) | 56.7% (38/67) | 65.1% (56/86) | 53.7% (51/95) | 44.4% (28/63) | 66.9% (79/118) |
| Explore spirituality or the sacred | 37.6% (68/181) | 35.2% (37/109) | 41.2% (28/68) | 25.0%  (1/4) | 26.7%  (16/60) | 43.0%  (52/121) | 35.1% (40/114) | 41.8% (28/67) | 43.0% (37/86) | 32.6% (31/95) | 23.8% (15/63) | 44.9% (53/118) |
| To help with mental health concerns | 57.5% (104/181) | 57.1% (60/109) | 61.8% (42/68) | 50.0%  (2/4) | 55.0%  (33/60) | 58.7%  (71/121) | 58.8% (67/114) | 55.2% (37/67) | 58.1% (50/86) | 56.8% (54/95) | 47.6% (30/63) | 62.7% (74/118) |
| **Time since last experience** | | | | | | | | | | | | |
| Within 24 hours | 8.8% (16/181) | 5.5%  (6/109) | 14.7% (10/68) | 0.0%  (0/4) | 5.0%  (3/60) | 10.7%  (13/121) | 10.5% (12/114) | 5.0%  (4/67) | 5.8%  (5/86) | 11.6% (11/95) | 4.8%  (3/63) | 11.0% (13/118) |
| Within the past week | 17.7% (32/181) | 15.6% (17/109) | 20.6% (14/68) | 25.0%  (1/4) | 16.7%  (10/60) | 18.2%  (22/121) | 13.2% (15/114) | 25.4% (17/67) | 20.9% (18/86) | 14.7% (14/95) | 9.5%  (6/63) | 22.0% (26/118) |
| Within the past month | 14.4% (26/181) | 15.6% (17/109) | 13.2% (9/68) | 0.0%  (0/4) | 11.7%  (7/60) | 15.7%  (19/121) | 15.8% (18/114) | 11.9% (8/67) | 11.6% (10/86) | 16.8% (16/95) | 17.5% (11/63) | 12.7% (15/118) |
| Within the past year | 30.9% (56/181) | 30.3% (33/109) | 20.9% (21/68) | 50.0%  (2/4) | 35.0%  (21/60) | 28.9%  (35/121) | 36.0% (41/114) | 22.4% (15/67) | 37.2% (32/86) | 25.3% (24/95) | 30.2% (19/63) | 31.4% (37/118) |
| In the past 5 years | 12.7% (23/181) | 13.7% (15/109) | 10.3% (7/68) | 25.0%  (1/4) | 16.7%  (10/60) | 10.7%  (13/121) | 14.9% (17/114) | 9.0%  (6/67) | 7.0%  (6/86) | 17.9% (17/95) | 15.9% (10/63) | 11.0% (13/118) |
| 5-10 years | 5.0%  (9/181) | 7.3%  (8/109) | 1.5%  (1/68) | 0.0%  (0/4) | 3.3%  (2/60) | 5.8%  (7/121) | 4.4%  (5/114) | 4.1%  (4/67) | 7.0%  (6/86) | 3.2%  (3/95) | 4.8%  (3/63) | 5.1%  (6/118) |
| More than 10 years | 7.7%  (14/181) | 9.2% (10/109) | 5.9%  (4/68) | 0.0%  (0/4) | 6.7%  (4/60) | 8.7%  (10/121) | 1.8%  (2/114) | 17.9% (12/67) | 7.0%  (6/86) | 8.4%  (8/95) | 11.1% (7/63) | 5.9%  (7/118) |
| **Did your past psychedelic use help with your mental health concerns?**^3^ | | | | | | | | | | | | |
| Yes | 68.6%(118/172) | 68.6%(72/105) | 69.8%(44/63) | 50.0%  (2/4) | 57.9%  (33/57) | 73.9%  (85/115) | 68.5%(76/111) | 68.9%(42/61) | 74.4%(61/82) | 63.3%(57/90) | 54.1%(33/61)^2^ | 76.6%(85/111) |
| No | 14.5%  (25/172) | 15.2%(16/105) | 14.3%  (9/63) | 0.0%  (0/4) | 17.5%  (10/57) | 13.0%  (15/115) | 16.2%(18/111) | 11.5%  (7/61) | 14.6%(12/82) | 14.4%(13/90) | 26.2%(16/61)^2^ | 8.1%  (9/111) |
| I don’t know/Not sure | 11.0%  (19/172) | 9.5%  (10/105) | 12.7%  (8/63) | 25.0%  (1/4) | 14.0%  (8/57) | 9.6%  (11/115) | 10.8%(12/111) | 11.5%  (7/61) | 6.1%  (5/82) | 15.6%(14/90) | 13.1%  (8/61)^2^ | 9.9%  (11/111) |
| Other | 5.8%  (10/172) | 6.7%  (7/105) | 3.2%  (2/63) | 25.0%  (1/4) | 10.5%  (6/57) | 3.5%  (4/115) | 4.5%  (5/111) | 8.2%  (5/61) | 4.9%  (4/82) | 6.7%  (6/90) | 6.6%  (4/61)^2^ | 5.4%  (6/111) |

^1^This was a multi-select question.

^2^Significant Chi-square tests (uncorrected p < .05), suggesting significant effects of the identified demographic factors on results.

^3^172 out of 181 participants who had experiences using psychedelics responded to this item.
