# Supplementary material for: Knowledge, perceptions, and use of psychedelics for mental health among autistic adults: An online survey
Source: PLOS Ment Health. 2025 Dec 26;2(12):e0000514. doi: 10.1371/journal.pmen.0000514 (PMC12798463; doi:10.1371/journal.pmen.0000514)
Supplement: S3 Table — (DOCX) [file pmen.0000514.s004.docx]

**Knowledge, Perceptions, and Use of Psychedelics for Mental Health among Autistic Adults: An Online Survey**

Sahba Afsharnia^1,2^, Vivian Liang^1,3^, Yona Lunsky^1,4^, Aaron P. Orsini^5^, Ami Tint^6^, Hsiang-Yuan Lin^1,2,4*^

**Supporting Information File: S3 Table**

S3 Table: Psychedelic experience that led to improvements in your mental health, stratified by sex, education, age, marital status, and country of residence

**S3 Table. Psychedelic experience that led to improvements in your mental health, stratified by sex, education, age, marital status, and country of residence**

|  | **Whole Sample**  **(N=147)** | **Sex** | | | **Education** | | **Age** | | **Marital Status** | | **Country of Residence** | |
| --- | --- | --- | --- | --- | --- | --- | --- | --- | --- | --- | --- | --- |
|  |  | **Assigned female at birth** | **Assigned male at birth** | **Other** | **Completion of Secondary School Education or less** | **Completion of Post-Secondary School Education** | **Age <40** | **Age ≥40** | **Single** | **Married/ Partnered** | **Canadian** | **Non- Canadian** |
| **Which of these mental health conditions improved after using psychedelics?^1^** | | | | | | | | | | | | |
| Anxiety Disorder | 76.9  (113/147) | 73.0%  (65/89) | 85.2%  (46/54) | 50.0%  (2/4) | 74.5%  (35/47) | 78.0%  (78/100) | 78.5%  (73/93) | 74.1%  (40/54) | 80.0%  (56/70) | 74.0%  (57/77) | 68.9%  (31/45) | 80.4%  (82/102) |
| Eating Disorder | 10.9% (16/147) | 10.1%  (9/89) | 13.0%  (7/54) | 0.0%  (0/4) | 14.9%  (7/47) | 9.0%  (9/100) | 16.1%  (15/93) | 1.9%  (1/54) | 10.0%  (7/70) | 11.7%  (9/77) | 4.4%  (2/45) | 13.7%  (14/102) |
| Impulse Control Disorder | 4.1%  (6/147) | 2.3%  (3/89) | 5.6%  (3/54) | 0.0%  (0/4) | 2.1%  (1/47) | 5.0%  (5/100) | 6.5%  (6/93) | 0.0%  (0/54) | 7.1%  (5/70) | 1.3%  (1/77) | 2.2%  (1/45) | 4.9%  (5/102) |
| Mood Disorder (Depression, Mania, Bipolar, etc.) | 56.5%  (83/147) | 55.1%  (49/89) | 59.3%  (32/54) | 50.0%  (2/4) | 48.9%  (23/47) | 60.0%  (60/100) | 60.2%  (56/93) | 50.0%  (27/54) | 50.0%  (35/70) | 62.3%  (48/77) | 55.6%  (25/45) | 56.9%  (58/102) |
| Post-traumatic Stress Disorder (PTSD) | 40.1%  (59/147) | 49.4%  (44/89) | 27.8%  (15/54) | 0.0%  (0/4) | 34.0%  (16/47) | 43.0%  (43/100) | 39.8%  (37/93) | 40.7%  (22/54) | 38.6%  (27/70) | 41.6%  (32/77) | 35.6%  (16/45) | 42.2%  (43/102) |
| Obsessive Compulsive Disorder (OCD) | 12.9%  (19/147) | 7.9%  (7/89) | 22.2%  (12/54) | 0.0%  (0/4) | 12.8%  (6/47) | 13.0%  (13/100) | 15.1%  (14/93) | 9.3%  (5/54) | 12.9%  (9/70) | 13.0%  (10/77) | 8.9%  (4/45) | 14.7%  (15/102) |
| Personality Disorder | 8.8%  (13/147) | 6.7%  (6/89) | 13.0%  (7/54) | 0.0%  (0/4) | 10.6%  (5/47) | 80%  (8/100) | 11.8%  (11/93) | 3.7%  (2/54) | 8.6%  (6/70) | 9.1%  (7/77) | 8.9%  (4/45) | 8.8%  (9/102) |
| Psychotic Disorder | 1.4%  (2/147) | 0.0%  (0/89) | 3.7%  (2/54) | 0.0%  (0/4) | 2.1%  (1/47) | 1.0%  (1/100) | 2.2%  (2/93) | 0.0%  (0/54) | 2.9%  (2/70) | 0.0%  (0/77) | 2.2%  (1/45) | 1.0%  (1/102) |
| Substance­ Related Disorder | 17.0% (25/147) | 12.4%  (11/89) | 24.1%  (13/54) | 25.0%  (1/4) | 23.4%  (11/47) | 14.0%  (14/100) | 18.3%  (17/93) | 14.8%  (8/54) | 17.1%  (12/70) | 16.9%  (13/77) | 17.8%  (8/45) | 16.7%  (17/102) |
| Neurodevelopmental Disorders | 33.3%  (49/147) | 32.6%  (29/89) | 37.0%  (20/54) | 0.0%  (0/4) | 40.4%  (19/47) | 30.0%  (30/100) | 36.6%  (34/93) | 27.8%  (15/54) | 30.0%  (21/70) | 36.4%  (28/77) | 28.9%  (13/45) | 35.3%  (36/102) |
| Burnout, Inertia, Meltdown, and Shutdown | 26.5%  (39/147) | 28.1%  (25/89) | 25.9%  (14/54) | 0.0%  (0/4) | 23.4%  (11/47) | 28.0%  (28/100) | 25.8%  (24/93) | 27.8%  (15/54) | 24.3%  (17/70) | 28.6%  (22/77) | 20.0%  (9/45) | 29.4%  (30/102) |
| Existential Crisis | 34.7%  (51/147) | 31.5%  (28/89) | 40.7%  (22/54) | 25.0%  (1/4) | 29.8%  (14/47) | 37.0%  (37/100) | 34.3%  (32/93) | 35.2%  (19/54) | 32.9%  (23/70) | 36.4%  (28/77) | 28.9%  (13/45) | 37.3%  (38/102) |
| Other | 11.6%  (17/147) | 11.2%  (10/89) | 9.3%  (5/54) | 50.0%  (2/4) | 14.9%  (7/47) | 10.0%  (10/100) | 9.7%  (9/93) | 14.8%  (8/54) | 7.1%  (5/70) | 15.6%  (12/77) | 17.8%  (8/45) | 8.8%  (9/102) |
| **Mean age at which the psychedelic experience that improved mental health took place** | 29.852 | 28.8  (SD = 10.227) | 31.3  (SD = 12.576) | 33.5  (SD = 12.793) | 28.6 (SD = 11.295) | 30.5  (SD = 11.210) | 25.0  (SD = 5.094) | 38.2  (SD = 13.717) | 30.5  (SD = 10.554) | 29.2  (SD = 11.891) | 30.0  (SD = 11.170) | 29.8  (SD = 11.318) |
| **How would you describe your mental health improvement after this psychedelic experience?** | | | | | | | | | | | | |
| Stopped experiencing the mental health concerns completely since the experience (full remission). | 2.7%  (4/146) | 1.1%  (1/89) | 5.7%  (3/53) | 0.0%  (0/4) | 2.1%  (1/47) | 3.0%  (3/99) | 2.2%  (2/93) | 3.8%  (2/53) | 2.9%  (2/69) | 2.6%  (2/77) | 4.4%  (2/45) | 2.0%  (2/101) |
| Greatly reduced experiencing the mental health concern(s) since the experience. | 32.2%  (47/146) | 27.0%  (24/89) | 39.6%  (21/53) | 50.0%  (2/4) | 31.9%  (15/47) | 32.3%  (32/99) | 31.2%  (29/93) | 34.0%  (18/53) | 37.7%  (26/69) | 27.3%  (21/77) | 24.4%  (11/45) | 35.6%  (36/101) |
| Reduced experiencing the mental health concern(s) somewhat since the experience. | 24.7%  (36/146) | 25.8%  (23/89) | 22.6%  (12/53) | 25.0%  (1/4) | 27.7%  (13/47) | 23.2%  (23/99) | 28.0%  (26/93) | 18.9%  (10/53) | 26.1%  (18/69) | 23.4%  (18/77) | 24.4%  (11/45) | 24.8%  (25/101) |
| Initially stopped experiencing the mental health concern(s) **completely**, then the mental health concern(s) returned to the same level as before. | 8.2%  (12/146) | 9.0%  (8/89) | 7.6%  (4/53) | 0.0%  (0/4) | 4.3%  (2/47) | 10.1%  (10/99) | 8.6%  (8/93) | 7.6%  (4/53) | 5.8%  (4/69) | 10.4%  (8/77) | 11.1%  (5/45) | 6.9%  (7/101) |
| Stopped experiencing the mental health concern(s) somewhat for a period of time, then the mental health concern(s) returned at the same level as before. | 16.4%  (24/146) | 27.0%  (24/89) | 13.2%  (7/53) | 0.0%  (0/4) | 17.0%  (8/47) | 16.2%  (16/99) | 17.2%  (16/93) | 15.1%  (8/53) | 11.6%  (8/69) | 20.8%  (16/77) | 15.6%  (7/45) | 16.8%  (17/101) |
| Other | 15.8%  (23/146) | 18.0%  (16/89) | 11.3%  (6/53) | 25.0%  (1/4) | 17.0%  (8/47) | 15.2%  (15/99) | 12.9%  (12/93) | 20.8%  (11/53) | 15.9%  (11/69) | 15.6%  (12/77) | 20.0%  (9/45) | 13.9%  (14/101) |
| **How long did your mental health improvement last?** | | | | | | | | | | | | |
| Less than 1 week | 9.6%  (14/146) | 10.1%  (9/89) | 9.4%  (5/53) | 0.0%  (0/4) | 12.8%  (6/47) | 8.1%  (8/99) | 8.6%  (8/93) | 11.3%  (6/53) | 10.1%  (7/69) | 9.1%  (7/77) | 20.0%  (9/45) | 5.0%  (5/101) |
| 1 -­ 2 weeks | 13.0%  (19/146) | 15.7%  (14/89) | 9.4%  (5/53) | 0.0%  (0/4) | 17.0%  (8/47) | 11.1%  (11/99) | 14.0%  (13/93) | 11.3%  (6/53) | 13.0%  (9/69) | 13.0%  (10/77) | 11.1%  (5/45) | 13.9%  (14/101) |
| 3 -­ 4 weeks | 6.8%  (10/146) | 5.6%  (5/89) | 7.6%  (4/53) | 25.0%  (1/4) | 6.4%  (3/47) | 7.1%  (7/99) | 8.6%  (8/93) | 3.8%  (2/53) | 5.8%  (4/69) | 7.8%  (6/77) | 6.7%  (3/45) | 6.9%  (7/101) |
| 1 -­ 3 months | 8.9%  (13/146) | 6.7%  (6/89) | 11.3%  (6/53) | 25.0%  (1/4) | 8.5%  (4/47) | 9.1%  (9/99) | 6.5%  (6/93) | 13.2%  (7/53) | 8.7%  (6/69) | 9.1%  (7/77) | 6.7%  (3/45) | 9.9%  (10/101) |
| 4 ­- 6 months | 15.1%  (22/146) | 18.0%  (16/89) | 9.4%  (5/53) | 25.0%  (1/4) | 14.9%  (7/47) | 15.2%  (15/99) | 15.1%  (14/93) | 15.1%  (8/53) | 14.5%  (10/69) | 15.6%  (12/77) | 15.6%  (7/45) | 14.9%  (15/101) |
| 7 -­ 12 months | 8.2%  (12/146) | 9.0%  (8/89) | 7.6%  (4/53) | 0.0%  (0/4) | 6.4%  (3/47) | 9.1%  (9/99) | 10.0%  (9/93) | 5.7%  (3/53) | 7.3%  (5/69) | 9.1%  (7/77) | 6.7%  (3/45) | 8.9%  (9/101) |
| 1 -­ 2 years | 17.1%  (25/146) | 15.7%  (14/89) | 20.8%  (11/53) | 0.0%  (0/4) | 17.0%  (8/47) | 17.2%  (17/99) | 19.4%  (18/93) | 13.2%  (7/53) | 17.4%  (12/69) | 16.9%  (13/77) | 17.8%  (8/45) | 16.8%  (17/101) |
| 3 -­ 5 years | 9.6  (14/146) | 11.2%  (10/89) | 7.6%  (4/53) | 0.0%  (0/4) | 8.5%  (4/47) | 10.1%  (10/99) | 9.7%  (9/93) | 9.4%  (5/53) | 8.7%  (6/69) | 10.4%  (8/77) | 6.7%  (3/45) | 10.9%  (11/101) |
| 6 -­ 10 years | 3.4%  (5/146) | 3.4%  (3/89) | 3.8%  (2/53) | 0.0%  (0/4) | 2.1%  (1/47) | 4.0%  (4/99) | 5.4%  (5/93) | 0.0%  (0/53) | 4.4%  (3/69) | 2.6%  (2/77) | 6.7%  (3/45) | 2.0%  (2/101) |
| 11 -­ 20 years | 4.1%  (6/146) | 2.2%  (2/89) | 7.6%  (4/53) | 0.0%  (0/4) | 2.1%  (1/47) | 5.1%  (5/99) | 3.2%  (3/93) | 5.7%  (3/53) | 7.3%  (5/69) | 1.3%  (1/77) | 0.0%  (0/45) | 5.9%  (6/101) |
| More than 20 years | 4.1%  (6/146) | 2.2%  (2/89) | 5.7%  (3/53) | 25.0%  (1/4) | 4.3%  (2/47) | 4.0%  (4/99) | 0.0%  (0/93) | 11.3%  (6/53) | 2.9%  (2/69) | 5.2%  (4/77) | 2.2%  (1/45) | 5.0%  (5/101) |
| **Which substance led to the psychedelic experience associated with your improvement in mental health concerns?** | | | | | | | | | | | | |
| psilocybin mushrooms | 55.5%  (81/146) | 58.4%  (52/89) | 46.1%  (26/53) | 75%  (3/4) | 61.7%  (29/47) | 52.5%  (52/99) | 55.9%  (52/93) | 54.7%  (29/53) | 52.2%  (36/69) | 58.4%  (45/77) | 68.9%  (31/45) | 49.5%  (50/101) |
| LSD | 15.1%  (22/146) | 13.5%  (12/89) | 17.0%  (9/53) | 25.0% (1/4) | 21.3%  (10/47) | 12.1%  (12/99) | 17.2%  (16/93) | 11.3%  (6/53) | 14.5%  (10/69) | 15.6%  (12/77) | 8.9%  (4/45) | 17.8%  (18/101) |
| morning glory seeds | 0/0%  (0/146) | 0.0%  (0/89) | 0.0%  (0/53) | 0.0% (0/4) | 0.0%  (0/47) | 0.0%  (0/99) | 0.0%  (0/93) | 0.0%  (0/53) | 0.0%  (0/69) | 0.0%  (0/77) | 0.0%  (0/45) | 0.0%  (0/101) |
| mescaline (pure compound) | 0/0%  (0/146) | 0.0%  (0/89) | 0.0%  (0/53) | 0.0% (0/4) | 0.0%  (0/47) | 0.0%  (0/99) | 0.0%  (0/93) | 0.0%  (0/53) | 0.0%  (0/69) | 0.0%  (0/77) | 0.0%  (0/45) | 0.0%  (0/101) |
| peyote cactus | 1.4%  (2/146) | 1.1%  (1/89) | 1.9%  (1/53) | 0.0% (0/4) | 0.0%  (0/47) | 2.0%  (2/99) | 2.2%  (2/93) | 0.0%  (0/53) | 2.9%  (2/69) | 0.0%  (0/77) | 2.2%  (1/45) | 1.0%  (1/101) |
| San Pedro cactus | 2.1%  (3/146) | 1.1%  (1/89) | 3.8%  (2/53) | 0.0% (0/4) | 0.0%  (0/47) | 3.0%  (3/99) | 3.2%  (3/93) | 0.0%  (0/53) | 2.9%  (2/69) | 1.3%  (1/77) | 0.0%  (0/45) | 3.0%  (3/101) |
| DMT (pure compound) | 2.7%  (4/146) | 0.0%  (0/89) | 7.6%  (4/53) | 0.0% (0/4) | 0.0%  (0/47) | 4.0%  (4/99) | 2.2%  (2/93) | 3.8%  (2/53) | 1.5%  (1/69) | 3.9%  (3/77) | 2.2%  (1/45) | 3.0%  (3/101) |
| Ayahuasca | 0.7%  (1/146) | 1.1%  (1/89) | 0.0%  (0/53) | 0.0% (0/4) | 0.0%  (0/47) | 1.0%  (1/99) | 1.1%  (1/93) | 0.0%  (0/53) | 0.0%  (0/69) | 1.3%  (1/77) | 0.0%  (0/45) | 1.0%  (1/101) |
| MDMA (ecstasy, Molly) | 13.0%  (19/146) | 14.6%  (13/89) | 11.3%  (6/53) | 0.0% (0/4) | 10.6%  (5/47) | 14.1%  (14/99) | 11.8%  (11/93) | 15.1%  (8/53) | 14.5%  (10/69) | 11.7%  (9/77) | 11.1%  (5/45) | 13.9%  (14/101) |
| Other | 9.6%  (14/146) | 10.1%  (9/89) | 9.4%  (5/53) | 0.0% (0/4) | 6.4%  (3/47) | 11.1%  (11/99) | 6.5%  (6/93) | 15.1%  (8/53) | 11.6%  (8/69) | 7.8%  (6/77) | 6.7%  (3/45) | 10.9%  (11/101) |
| **Psychedelic doses improving mental health issues in those with positive experiences (n=146)** | | | | | | | | | | | | |
| Very low | 9.6%  (14/146) | 9.0%  (8/89)^2^ | 9.4%  (5/53) | 25.0% (1/4)^2^ | 14.9%  (7/47) | 7.1%  (7/99) | 7.5%  (7/93) | 13.2% (7/53) | 7.4%  (5/69) | 11.7%  (9/77) | 13.3%  (6/45) | 7.9%  (8/101) |
| Low | 10.3% (15/146) | 11.2% (10/89)^2^ | 7.5%  (4/53) | 25.0% (1/4)^2^ | 4.3%  (2/47) | 17.2%  (17/99) | 10.8% (10/93) | 9.4%  (5/53) | 5.9%  (4/69) | 14.3% (11/77) | 11.1%  (5/45) | 9.9% (10/101) |
| Moderate | 32.9% (48/146) | 40.4% (36/89)^2^ | 20.8% (11/53) | 25.0% (1/4)^2^ | 40.4%  (19/47) | 28.3%  (28/99) | 39.8% (37/93) | 20.8% (11/53) | 31.9% (22/69) | 32.5% (25/77) | 26.7% (12/45) | 35.6% (36.101) |
| High | 13.7% (20/146) | 7.9% (7/89)^2^ | 24.5% (13/53) | 0.0% (0/4)^2^ | 6.4%  (3/47) | 17.2%  (17/99) | 10.8  (10/93) | 18.87% (10/53) | 18.8% (13/69) | 9.1%  (7/77) | 11.1% (5/45) | 14.9% (15/101) |
| Very high | 8.9% (13/146) | 9.0% (8/89)^2^ | 7.5%  (4/53) | 25.0% (1/4)^2^ | 10.6%  (5/47) | 8.1%  (8/99) | 9.7% (9/93) | 7.5% (4/53) | 8.7%  (6/69) | 9.1%  (7/77) | 8.9%  (4/45) | 8.9% (9/101) |
| **Location of psychedelic experience that helped with mental health concern^1^** | | | | | | | | | | | | |
| Home | 63.9% (94/147) | 66.3% (59/89) | 59.3% (32/54) | 75.0% (3/4) | 74.5%  (35/47) | 59.0%  (59/100) | 67.7% (63/93) | 57.4% (31/54) | 55.7% (39/70) | 71.4% (55/77) | 68.9% (31/45) | 61.8% (63/102) |
| Party | 5.4%  (8/147) | 6.7% (6/89) | 3.7%  (2/54) | 0.0% (0/4) | 8.5%  (4/47) | 4.0%  (4/100) | 4.3% (4/93) | 7.4% (4/54) | 4.3% (3/70) | 6.5%  (5/77) | 6.7%  (3/45) | 4.9% (5/102) |
| Public place | 3.4%  (5/147) | 4.5% (4/89) | 1.9%  (1/54) | 0.0% (0/4) | 4.2%  (2/47) | 3.0%  (3/100) | 4.3% (4/93) | 1.9% (1/54) | 2.9% (2/70) | 3.9%  (3/77) | 6.7%  (3/45) | 2.0% (2/102) |
| Concert | 9.5% (14/147) | 9.0% (8/89) | 9.3%  (5/54) | 25.0% (1/4) | 8.5%  (4/47) | 10.0%  (10/100) | 8.6% (8/93) | 11.1% (6/54) | 7.1% (5/70) | 11.7% (9/77) | 8.9%  (4/45) | 9.8% (10/102) |
| Nature | 33.3% (49/147) | 36.0% (32/89) | 25.9% (14/54) | 75.0% (3/4) | 31.9%  (15/47) | 34.0%  (34/100) | 36.6% (34/93) | 27.8% (15/54) | 31.4% (22/70) | 35.1% (27/77) | 31.1% (14/45) | 34.3% (35/102) |
| Religious | 7.5% (11/147) | 7.9% (7/89) | 7.4%  (4/54) | 0.0% (0/4) | 6.4%  (3/47) | 8.0%  (8/100) | 8.6% (8/93) | 5.6% (3/54) | 8.6% (6/70) | 6.5%  (5/77) | 2.2%  (1/45) | 9.8% (10/102) |
| Other | 8.8% (13/147) | 9.0% (8/89) | 7.4%  (4/54) | 25.0% (1/4) | 8.5%  (4/47) | 9.0%  (9/100) | 4.3% (4/93) | 16.7% (9/54) | 15.7  (11/70) | 2.6%  (2/77) | 13.3% (6/45) | 6.9% (7/102) |

^1^This was a multi-select question.

^2^Significant Chi-square tests (uncorrected p < .05), suggesting significant effects of the identified demographic factors on results.
