# Supplementary material for: Knowledge, perceptions, and use of psychedelics for mental health among autistic adults: An online survey
Source: PLOS Ment Health. 2025 Dec 26;2(12):e0000514. doi: 10.1371/journal.pmen.0000514 (PMC12798463; doi:10.1371/journal.pmen.0000514)
Supplement: S4 Table — (DOCX) [file pmen.0000514.s005.docx]

**Knowledge, Perceptions, and Use of Psychedelics for Mental Health among Autistic Adults: An Online Survey**

Sahba Afsharnia^1,2^, Vivian Liang^1,3^, Yona Lunsky^1,4^, Aaron P. Orsini^5^, Ami Tint^6^, Hsiang-Yuan Lin^1,2,4*^

**Supporting Information File: S4 Table**

S4 Table: Feelings, thoughts, and experiences during the overall psychedelic experience that led to improvements in your mental health, stratified by sex, education, age, marital status, and country of residence

**S4 Table: Feelings, thoughts, and experiences during the overall psychedelic experience that led to improvements in your mental health, stratified by sex, education, age, marital status, and country of residence**

|  | **Whole Sample**  **(N=181)** | **Sex** | | | **Education** | | **Age** | | **Marital Status** | | **Country of Residence** | |
| --- | --- | --- | --- | --- | --- | --- | --- | --- | --- | --- | --- | --- |
|  |  | **Assigned female at birth** | **Assigned male at birth** | **Other** | **Completion of Secondary School Education or less** | **Completion of Post-Secondary School Education** | **Age <40** | **Age ≥40** | **Single** | **Married/ Partnered** | **Canadian** | **Non- Canadian** |
| **How personally meaningful was the psychedelic experience that led to mental health improvement?** | | | | | | | | | | | | |
| Low meaningfulness (“not meaningful at all” or “slightly meaningful”) | 8.9%  (13/146) | 7.87% (7/89) | 11.3% (6/53) | 0.0% (0/4) | 6.4%  (3/47) | 10.1%  (10/99) | 7.5%  (7/93) | 11.3%  (6/53) | 8.7%  (6/69) | 9.1%  (7/77) | 15.6%  (7/45) | 5.9%  (6/101) |
| Moderate meaningfulness (“mildly meaningful” or “moderately meaningful”) | 38.4%  (56/146) | 43.8% (39/89) | 26.4% (14/53) | 75.0% (3/4) | 29.8%  (14/47) | 42.4%  (42/99) | 41.9%  (39/93) | 32.1%  (17/53) | 34.8%  (24/69) | 41.6%  (32/77) | 44.4%  (20/45) | 35.6%  (36/101) |
| High meaningfulness (“strongly meaningful” or “extremely meaningful”) | 52.7%  (77/146) | 48.3% (43/89) | 62.3% (33/53) | 25.0% (1/4) | 63.9%  (30/47) | 47.5%  (47/99) | 50.5%  (47/93) | 56.6%  (30/53) | 56.5%  (39/69) | 49.4%  (38/77) | 40.0%  (18/45) | 58.4%  (59/101) |
| **Degree to which the psychedelic experience that led to your improvement in mental health issues were spiritually significant to you.**^2^ | | | | | | | | | | | | |
| Low significance (“not at all” or “slightly”) | 31.0%  (45/145) | 30.7% (27/88) | 41.5% (22/53) | 25.0% (1/4) | 30.4%  (14/46) | 31.3%  (31/99) | 31.5%  (29/92) | 30.2%  (16/53) | 30.4%  (21/69) | 31.6%  (24/76) | 37.8%  (17/45) | 28.0% (28/100) |
| Moderate significance (“moderately” or “very much”) | 37.9%  (55/145) | 37.5% (33/88) | 35.8% (19/53) | 75.0% (3/4) | 34.8%  (16/46) | 39.4%  (39/99) | 35.9%  (33/92) | 41.5%  (22/53) | 33.3%  (23/69) | 42.1%  (32/76) | 22.2%  (10/45) | 37.0% (37/100) |
| High significance (“among the 5 most spiritually significant experiences of my life” or “the single most spiritually significant experience of my life”) | 31.0%  (45/145) | 31.8% (28/88) | 32.1% (17/53) | 0.0% (0/4) | 34.8%  (16/46) | 29.3%  (29/99) | 32.6%  (30/92) | 28.3% (15/53) | 36.2%  (25/69) | 26.3%  (20/76) | 40.0%  (18/45) | 35.0% (35/100) |
| **How psychologically challenging was the psychedelic experience that led to improvements in your mental health concerns?**^2^ | | | | | | | | | | | | |
| Low challenge (“not challenging at all” or “slightly challenging”) | 37.2%  (54/145) | 39.8% (35/88)^6^ | 34.0% (18/53) | 25.0% (1/4) | 32.6%  (15/46) | 39.4%  (39/99) | 35.9%  (33/92) | 41.5%  (22/53) | 37.7%  (26/69) | 27.6%  (21/76) | 42.2%  (19/45) | 35.0% (33/100) |
| Moderate challenge (“mildly challenging” or “moderately challenging”) | 43.4%  (63/145) | 47.7% (42/88)^6^ | 34.0% (18/53) | 75.0% (3/4) | 45.7%  (21/46) | 42.4%  (42/99) | 44.6%  (41/92) | 39.6%  (21/53) | 40.6%  (28/69) | 46.1%  (35/76) | 42.2%  (19/45) | 44.0% (44/100) |
| High challenge (“strongly challenging” or “extremely challenging”) | 19.3%  (28/145) | 12.5% (11/88)^6^ | 32.1% (17/53) | 0.0% (0/4) | 21.7%  (10/46) | 18.2%  (18/99) | 19.6%  (18/92) | 18.9%  (10/53) | 21.7%  (15/69) | 17.1%  (13/76) | 15.6%  (7/45) | 21.0% (21/100) |
| **How psychologically insightful to you was the psychedelic experience that led to your improvement in mental health concerns?** | | | | | | | | | | | | |
| Low insight (“not psychologically insightful at all” or slightly psychologically insightful”) | 22.8%  (33/145) | 11.4% (10/88)^6^ | 11.3% (6/53) | 0.0% (0/4) | 8.7%  (4/46) | 12.2%  (12/99) | 9.8%  (9/92) | 13.2%  (7/53) | 10.1%  (7/69) | 11.8%  (9/76) | 20.0%  (9/45) | 7.0% (7/100) |
| Moderate insight (“mildly insightful” or “moderately insightful”) | 21.4%  (31/145) | 38.6% (34/88)^6^ | 18.9% (10/53) | 100.0% (4/4) | 28.3%  (13/46) | 35.4%  (35/99) | 39.1%  (36/92) | 22.6%  (12/53) | 34.8%  (24/69) | 22.4%  (17/76) | 28.9%  (13/45) | 35.0% (35/100) |
| High insight (“strongly psychologically insightful” or extremely psychologically insightful”) | 55.9%  (81/145) | 50.0% (44/88)^6^ | 69.8% (37/53) | 0.0% (0/4) | 63.0%  (29/46) | 52.5%  (52/99) | 51.1%  (47/92) | 64.2%  (34/53) | 55.1%  (38/69) | 56.6%  (43/76) | 51.1%  (23/45) | 58.0% (58/100) |
| **Items related to your psychedelic associated improvement in mental health concerns.**^2,5^ | | | | | | | | | | | | |
| Strengthening your belief in your own ability to recover | 67.6%  (98/145) | 68.2% (60/88) | 66.0% (35/53) | 75.0% (3/4) | 69.6%  (32/46) | 66.7%  (66/99) | 62.0%  (57/92) | 77.4%  (41/53) | 62.3%  (43/69) | 72.4%  (55/76) | 57.8%  (26/45) | 72.0% (72/100) |
| Reducing stress involved with recovering. | 62.1%  (90/145) | 65.9% (58/88) | 58.5% (31/53) | 25.0% (1/4) | 63.0%  (29/46) | 61.6%  (61/99) | 65.2%  (60/92) | 56.6%  (30/53) | 62.3%  (43/69) | 61.8%  (47/76) | 60.0%  (27/45) | 63.0% (63/100) |
| Reframing mental health improvement as a spiritual task. | 46.2%  (67/145) | 46.6% (41/88) | 43.4% (23/53) | 75.0% (3/4) | 45.7%  (21/46) | 46.5%  (46/99) | 43.5%  (40/92) | 50.9%  (27/53) | 47.8%  (33/69) | 44.7%  (34/76) | 31.1%  (14/45) | 53.0% (53/100) |
| Changing life priorities or values. | 60.7%  (88/145) | 59.1% (52/88) | 62.3% (33/53) | 75.0% (3/4) | 65.2%  (30/46) | 58.6%  (58/99) | 56.5%  (53/92) | 66.0%  (35/53) | 63.8%  (44/69) | 57.9%  (44/76) | 53.3%  (24/45) | 64.0% (64/100) |
| Changing your orientation toward the future, so that long­term benefits outweighed immediate desires/preoccupations. | 46.9%  (68/145) | 48.9% (43/88) | 47.2% (25/53) | 25.0% (1/4) | 45.7%  (21/46) | 47.5%  (47/99) | 50.0%  (46/92) | 41.5%  (22/53) | 46.4%  (32/69) | 47.4%  (36/76) | 51.1%  (23/45) | 45.0% (45/100) |
| **Other behavioural changes after the psychedelic experience** ^2,5^ | | | | | | | | | | | | |
| None | 30.3% (44/145) | 30.7% (27/88) | 30.2% (16/53) | 25.0% (1/4) | 30.4%  (14/46) | 30.3%  (30/99) | 29.3%  (27/92) | 32.1%  (17/53) | 29.0%  (20/69) | 31.6%  (24/76) | 55.6%  (25/45) | 19.0% (19/100) |
| Reduced/quit other drugs | 30.3% (44/145) | 26.1% (23/88) | 35.8% (19/53) | 50.0% (2/4) | 32.6%  (15/46) | 29.3%  (29/99) | 34.8%  (32/92) | 22.6%  (12/53) | 34.8%  (24/69) | 26.3%  (20/76) | 26.7%  (12/45) | 32.0% (32/100) |
| Started using other drugs | 6.2%  (9/145) | 5.7% (5/88) | 7.5% (4/53) | 0.0% (0/4) | 10.9%  (5/46) | 4.0%  (4/99) | 6.5%  (6/92) | 5.7%  (3/53) | 2.9%  (2/69) | 9.2%  (7/76) | 8.9%  (4/45) | 5.0% (5/100) |
| Changes in diet | 24.1%  (35/145) | 23.9% (21/88) | 26.4% (14/53) | 0.0% (0/4) | 30.4%  (14/46) | 21.2%  (21/99) | 22.8%  (21/92) | 26.4%  (14/53) | 27.5%  (19/69) | 21.1%  (16/76) | 15.6%  (7/45) | 28.0% (28/100) |
| Increased exercise | 31.0% (45/145) | 28.4% (25/88) | 35.8% (19/53) | 25.0% (1/4) | 28.3%  (13/46) | 32.3%  (32/99) | 32.6%  (30/92) | 28.3%  (15/53) | 36.2%  (25/69) | 26.3%  (20/76) | 35.6%  (16/45) | 29/0% (29/100) |
| Decreased exercise | 2.8%  (4/145) | 3.4% (3/88) | 1.9% (1/53) | 0.0% (0/4) | 4.3%  (2/46) | 2.0%  (2/99) | 2.2%  (2/92) | 3.8%  (2/53) | 1.4%  (1/69) | 3.9%  (3/76) | 4.4%  (2/45) | 2.0% (2/100) |
| Improved relationships | 63.4%  (92/145) | 63.6% (56/88) | 64.2% (34/53) | 50.0% (2/4) | 71.7%  (33/46) | 59.6%  (59/99) | 68.5%  (63/92) | 54.7%  (29/53) | 65.2%  (45/69) | 61.8%  (47/76) | 57.8%  (26/45) | 66.0% (66/100) |
| Worsened relationships | 5.5%  (8/145) | 8.0% (7/88) | 1.9% (1/53) | 0.0% (0/4) | 10.9%  (5/46) | 3.0%  (3/99) | 7.6%  (7/92) | 1.9%  (1/53) | 7.2%  (5/69) | 3.9%  (3/76) | 13.3%  (6/45) | 2.0% (2/100) |
| Improved career | 30.3%  (44/145) | 30.7% (27/88) | 30.2% (16/53) | 25.0% (1/4) | 32.6%  (15/46) | 29.3%  (29/99) | 35.9%  (33/92) | 20.8%  (11/53) | 34.8%  (24/69) | 26.3%  20/76) | 17.8%  (8/45) | 36.0% (36/100) |
| Worsened career | 4.8%  (7/145) | 6.8% (6/88) | 1.9% (1/53) | 0.0% (0/4) | 13.0%  (6/46) | 1.0%  (1/99) | 6.5%  (6/92) | 1.9%  (1/53) | 2.9%  (2/69) | 6.6%  (5/76) | 6.7%  (3/45) | 4.0% (4/100) |
| **Did you experience any persisting negative effects from this psychedelic experience?**^3^ | | | | | | | | | | | | |
| Yes | 10.0%  (17/170) | 9.6% (10/104) | 11.3% (7/62) | 0.0% (0/4) | 12.5%  (7/56) | 8.8%  (10/114) | 10.0%  (11/110) | 10.0%  (6/60) | 12.3%  (10/81) | 7.9%  (7/89) | 16.4%  (10/61) | 6.4%  (7/109) |
| No | 81.8% (139/170) | 82.7% (86/104) | 80.6% (50/62) | 75.0% (3/4) | 80.4%  (45/56) | 82.5%  (94/114) | 80.9%  (89/110) | 83.3%  (50/60) | 81.5%  (66/81) | 82.0%  (73/89) | 73.8%  (45/61) | 86.2%  (94/109) |
| Not sure | 8.2%  (14/170) | 7.7% (8/104) | 8.1% (5/62) | 25.0% (1/4) | 7.1%  (4/56) | 8.8%  (10/114) | 9.1%  (10/110) | 6.7%  (4/60) | 6.2%  (5/81) | 10.1%  (9/89) | 9.8%  (6/61) | 7.3%  (8/109) |
| **Overall, how would you rate the severity of these negative effects?^4^** | | | | | | | | | | | | |
| Low (“not at all severe” or “slightly severe”) | 87.5%  (147/168) | 87.3% (89/102) | 87.1% (54/62) | 100.0% (4/4) | 89.1%  (49/55) | 86.7%  (98/113) | 89.8%  (97/108) | 83.3%  (50/60) | 85.0% (68/80) | 89.8%  (79/88) | 82.0%  (50/61) | 90.7%  (97/107) |
| Moderate (“moderately severe”) | 8.9%  (15/168) | 9.8% (10/102) | 8.1% (5/62) | 0.0% (0/4) | 7.3%  (4/55) | 9.7%  (11/113) | 6.5%  (7/108) | 13.3%  (8/60) | 11.3%  (9/80) | 6.8%  (6/88) | 9.8%  (6/61) | 8.4%  (9/107) |
| High (“very severe” or “extremely severe”) | 3.6%  (6/168) | 2.9% (3/102) | 4.8% (3/62) | 0.0% (0/4) | 3.6%  (2/55) | 3.5%  (4/113) | 3.7%  (4/108) | 3.3%  (2/60) | 3.8% (3/80) | 3.4%  (3/88) | 8.2%  (5/61) | 0.9% (1/107) |

^1^Items in these tables were branched out questions, thus only 146 out of 181 participants who had experiences using psychedelics reported.

^2^1 out of 146 participants specified in the specified 1 did not respond to these items.

^3^170 out of 181 participants who had experiences using psychedelics responded to this item.

^4^2 out of 170 participants who reported negative experiences using psychedelics did not respond to this item.

^5^This was a multi-select question.

^6^Significant Chi-square tests (uncorrected p < .05), suggesting significant effects of the identified demographic factors on results.
