# Supplementary material for: Knowledge, perceptions, and use of psychedelics for mental health among autistic adults: An online survey
Source: PLOS Ment Health. 2025 Dec 26;2(12):e0000514. doi: 10.1371/journal.pmen.0000514 (PMC12798463; doi:10.1371/journal.pmen.0000514)
Supplement: Attachment — Submitted filename: Response to Reviewers_30Oct2025.docx [file pmen.0000514.s007.docx]

**Editor’s Comments**

**Comment 1:** Please amend your detailed Financial Disclosure statement. This is published with the article. It must therefore be completed in full sentences and contain the exact wording you wish to be published.

i. State the initials, alongside each funding source, of each author to receive each grant.

**Response:** We thank the editor for this clarification. The Financial Disclosure statement has been revised to ensure it is composed entirely of full sentences and to specify the initials of the authors associated with each funding source, in accordance with journal guidelines (see Page 26, lines 592–597 of the manuscript).

**Comment 2:** Please ensure that your Ethics Statement is available in its entirety at the beginning of your Methods section, under a subheading 'Ethics Statement'.

**Response:** We thank the Editor for this reminder. We have added a full Ethics Statement at the beginning of the Methods section under the subheading “Ethics Statement,” following the journal’s instructions (Page 5, lines 113–119 of the manuscript).

**Comment 3:** Tables should not be uploaded as individual files. Please remove these files and include the Tables in your manuscript file as editable, cell-based objects. For more information about how to format tables, see our guidelines:

[https://journals.plos.org/mentalhealth/s/tables](https://urldefense.com/v3/__https:/journals.plos.org/mentalhealth/s/tables__;!!FxkXuJIC!fE2RUyR_iBI9jmwwnb6O698o52nSA0rUN0MIOWBpjrGvdc7E5Z5WTUUim_yrQ8iB6C-AS6zu1fV8ReyaCTRcFQ$)

**Response:** We thank the Editor for this note. We have integrated all tables directly into the main manuscript file as editable, cell-based objects, in accordance with PLOS formatting guidelines (see Table 1 on p. 7; Table 2 on p. 9; Table 3 on p. 11; Table 4 on p. 14; Table 5 on p. 17). Separate table uploads have been removed.

**Comment 4:** We have noticed that you have uploaded Supporting Information files, but you have not included a list of legends. Please add a full list of legends for your Supporting Information files after the references list.

**Response:** We thank the Editor for identifying this oversight. A list of legends for the Supporting Information files has been added after the References section (see Page 30, lines 734–747).

**Reviewers’ Comments**

**Reviewer #1:** This manuscript presents a comprehensive cross-sectional survey exploring autistic adults’ knowledge, perceptions, and experiences with psychedelics. It addresses a significant gap in the literature by focusing on a marginalized and underrepresented group in clinical psychedelic research. The work is grounded in a strong rationale, methodologically rigorous, and offers novel insights relevant to both clinical research and public health.
see the attached for more details

**Response:** We sincerely thank the reviewer for the positive evaluation of our manuscript and the detailed, constructive recommendations provided in the attached file. We have addressed these specific points below.

**Comment 1:** **Clarify the role of self-diagnosis:** Present a more critical discussion on how self-diagnosed vs. clinically diagnosed groups may differ in interpretation of psychedelic experiences.

**Response:** We thank the reviewer for this important suggestion. We have added a new paragraph to the Discussion section (p. 23, L460–468) analyzing the similarities and differences between self-diagnosed (n=51) and clinically diagnosed (n=206) participants. We noted that the only statistically significant difference was in dosing practices (Table 3; p. 11, L255), while knowledge, perceptions, and interpretations of psychedelic experiences were largely similar (Tables 2, 4, 5). We discuss the implications of including self-diagnosed individuals, acknowledging the trade-off between inclusivity and diagnostic certainty, while noting the overall consistency in responses within this sample.

**Comment 2: Strengthen discussion on negative effects:** Expand the limited items assessing adverse outcomes; propose more robust monitoring in future studies.

**Response:** We appreciate the reviewer highlighting the need for a stronger discussion on adverse outcomes. We have revised the Discussion (p. 25, L533–541) to explicitly acknowledge that the assessment of adverse effects was limited (only three items). We also added specific recommendations for future research, emphasizing the need for structured monitoring and validated instruments to systematically capture the frequency, severity, and duration of potential negative outcomes, especially given the vulnerabilities of the autistic population.

**Comment 3: Address sample limitations:** Discuss how future research could better include BIPOC, non-English speakers, and those with greater support needs.

**Response:** We thank the reviewer for this recommendation. We have expanded the Limitations section (p. 24, L500-507 & L518-525) to include specific strategies for improving diversity in future research. We now suggest targeted outreach, partnering with organizations serving BIPOC communities, providing translated materials, and utilizing accessible study designs (e.g., non-text-based methods or facilitated interviews) to better include individuals with communication differences or greater support needs.

**Comment 4:** **Improve statistical reporting:** Acknowledge multiple comparisons and justify not applying correction (or apply it). Report effect sizes where relevant.

**Response:** We thank the reviewer for this statistical guidance. We have revised the Data Analysis section (p. 7, L193–196) to acknowledge the increased risk of Type I error due to multiple comparisons and to justify the use of uncorrected p-values based on the exploratory nature of the study. Furthermore, we have calculated and reported effect sizes (Phi or Cramer’s V) for all significant chi-square tests in the Secondary Analyses section (p. 20, L349–364 & p. 21, L361–369) and the Effects of Demographics section (p. 20, L322–340). During this process, we also corrected the reporting of the pairwise comparisons for meaningfulness (p. 21, L372–375).

**Comment 5:** **Tonal moderation:** Soften advocacy language in discussion/conclusion unless directly supported by the data or previous trial results.

**Response:** We appreciate the reviewer’s feedback regarding the tone of the manuscript. We have reviewed the Discussion and Conclusion and moderated the language to ensure interpretations do not overstate the findings. For example, we replaced "urgency of continued exploration" with "importance of continued exploration" in the Conclusion (p. 26, L561).

**Comment 6:** **Data access and replication:** Provide more detail on the OSF dataset and encourage secondary analyses.

**Response:** We thank the reviewer for supporting open science practices. We have updated the Data Availability Statement (p. 26, L600–601). to provide more detail, confirming that the anonymized dataset and the survey instrument (codebook) are available in the OSF repository. We also added a sentence explicitly encouraging secondary analyses of the dataset.

**Reviewer #2:** In general this was a well-written and designed paper focussed on an interesting and novel area. I only have comments regarding minor changes.

**Comment 1:** Data Availability. Authors link an OSF repository and state the data from the study is available here: [https://osf.io/zceyk/](https://urldefense.com/v3/__https:/osf.io/zceyk/__;!!FxkXuJIC!fE2RUyR_iBI9jmwwnb6O698o52nSA0rUN0MIOWBpjrGvdc7E5Z5WTUUim_yrQ8iB6C-AS6zu1fV8RewzO_ntdA$) however on accessing the link there is no data to download. I have still selected "Yes" in answer to question 3 above because the authors also include supplementary tables with survey responses in word format. Please remedy the OSF link issue.

**Response:** We thank the reviewer for catching this issue. We have verified and updated the Open Science Framework (OSF) repository link to ensure the full dataset is publicly accessible. The Data Availability Statement has been updated accordingly (p. 26, L600–601).

**Comment 2:** Abstract. Related to comments below regarding the specificity of this sample and the generalisability of the conclusions, I recommend specifying the conclusions may not apply to all autistic adults e.g., “Autistic adults *in this self-selecting sample* demonstrated strong interest...". (lines 37-38, p. 2).

**Response:** We thank the reviewer for this helpful suggestion. We have revised the Abstract (p. 2, L42) to specify that conclusions apply to this self-selecting sample to better reflect the study’s generalizability limitations.

Comment 3: Introduction. Very clear and generally well-written. Only minor recommendations:
a. "Ayahuasca" should not be capitalised (line 62, p. 3).

**Response:** We thank the reviewer for noting this. We have corrected the capitalization of ayahuasca throughout the manuscript (p. 3, L69).

b. Rephrase for English: “Only a pilot trial in modern-era research explored the use of MDMA in a small sample of autistic adults (N = 12; 8 in the MDMA group), demonstrating a promising signal of MDMA’s reducing social anxiety and good safety and tolerability (32).” (lines 78-80, p. 3). I know what you mean but needs to be rephrased.

**Response:** We thank the reviewer for this comment. We have rephrased the MDMA pilot-trial sentence (p. 4, L86-88) for improved clarity and readability in English.

c. I recommend including reference to negative side effects from these early studies for balance. Significant negative effects were also reported. See Markopoulos (2022), Bender et al. (1966) – e.g. aggression, biting, self-harm etc.

**Response:** We thank the reviewer for this important point. We added a balanced discussion acknowledging adverse effects observed in early studies (Bender et al., 1966; Markopoulos, 2022) to provide a more complete perspective (p. 4, L86-88).

d. “However, outcomes varied significantly based on individual mindsets, knowledge, and expectations.” This statement is not supported by the referenced study. (lines 86-87, p. 4). Either change the statement or provide a reference that supports it.

**Response:** We thank the reviewer for highlighting this. We confirm that the sentence (p. 4, L97) was revised to include appropriate references (Carhart-Harris & Friston, 2019) and ensure the phrasing accurately reflects the literature.

e. “Also, whether this first survey results are reliable in different autistic cohorts remains unknown.” Rephrase for English. E.g. “the results of this first survey” reads better than “this first survey results”. (lines 90-91, p. 4)

**Response:** We thank the reviewer. We corrected the minor grammatical issue (p. 4, L100–101) for smoother phrasing.

f. “(1) What are the general perspectives and knowledge levels of autistic adults on psychedelics?” – rephrase for English. (lines 94-95 p.4), “knowledge levels of autistic adults on psychedelics” does not read well.

**Response:** We thank the reviewer for noting this. We rephrased research question 1 for grammatical clarity (p. 4, L106–107).

g. In your second research question please clarify which type of symptoms you are looking for opinions on – autistic symptoms? Mental health condition symptoms? Both?

**Response:** We thank the reviewer for this useful clarification. In alignment with the revised scope of the study (see response to Reviewer 4), we clarified research question 2 to specify that we examined opinions on treating co-occurring mental-health symptoms (p. 4, L107–108).

**Comment 4:** Materials and Methods. Great and clear section. Only minor recommendations:
a. Remove additional ‘were’ from this phrasing “2) self-identified or were professionally diagnosed with ASC,” (lines 102-103, p. 4)

**Response:** We thank the reviewer. We removed the redundant “were” from the phrasing (p. 4, L123).

b. Specify which social media sites and community organisations you recruited from to better describe the sample. This can be very meaningful in online surveys. For example were the participants recruited from autism advocacy organisations? Were they recruited from psychedelics advocacy organisations or subreddits? This is an important descriptor for potential bias in the sample. Especially if in the discussion or abstract it is implied these findings may apply to the autistic population as a whole.

**Response:** We thank the reviewer for this valuable suggestion. The Methods section has been expanded to provide a detailed description of the recruitment sources, consistent with the approved REB protocol (REB #110-2022). We now specify that participants were recruited through social-media platforms, national autism and neurodevelopmental organizations, and psychedelic community networks across Canada (p. 4, L125-133).

**Comment 5:** Results. Clear and well-written. Only one recommendation: for concision and lack of repetition of data in the tables you could cut some of the main text and reserve it for highlighting only the most significant findings in the tables.

**Response:** We thank the reviewer for this valuable suggestion. The Results section (pp. ??-??) has been extensively edited for concision by removing text that repeats numerical data already presented in the tables. The revised section now focuses on highlighting only the most significant findings and trends while directing the reader to the tables for detailed statistics (e.g., several paragraphs were removed from p. 9, L236 & p. 11, L369, where they were originally located within the Results section. Some paragraphs were also shortened (see p. 6, L208-211 & p. 17, L280-286 & p. 17, L293-394)).

**Comment 6:** Discussion. Very clear, well-written and interesting. Only minor recommendations:
a. Most importantly, this paper needs more discussion around the possibility (probability) that the sample is significantly biased. I recommend this is also highlighted in the Abstract. For example, this was not a psychedelic naive sample (70% having used a psychedelic previously). Moreover, purely by the nature of the topic of survey (psychedelics) respondents may be more likely to answer if they already have an interest in the area. These findings may not generalise to the wider autistic population and this needs to be highlighted in the paper as a key limitation.

**Response:** We thank the reviewer for this important observation. We expanded the *Discussion* to explicitly address potential sampling bias arising from self-selection and the high proportion of participants with prior psychedelic experience (70%). We specifically noted that recruitment through psychedelic community networks likely contributed to this bias (p. 24, L 500-507). We also added a clarifying line to the Abstract noting that our sample may not be representative of the broader autistic population (p. 2, L46-47).

b. Relatedly, the following statement, "In terms of public perceptions, while the neurotypical population may harbor legal and safety concerns (37), our participants showed more willingness to engage in research, suggesting that autistic adults may be more open to alternative mental health treatments." (lines 361-363, p. 10) seems to imply that autism explains greater openness to alternative MH treatments, whilst it may in fact be due to the fact you have sampled a group of pro-psychedelic participants. Either modify the statement or provide evidence that it is in fact autism and not other factors (e.g. previous psychedelic use or recruitment from psychedelic related websites or organisations) causing this.

**Response:** We thank the reviewer for this thoughtful comment. We revised the statement (p. 22, L407-411) to clarify that the observed openness to psychedelic research could reflect characteristics of our self-selecting sample (e.g., prior psychedelic use or favorable attitudes), unique perspectives within the autistic community, or both. We also note that this distinction cannot be determined based on the current study design.

c. Discussion of the limitations of self-reported dosage in online surveys is also needed in addition to the limitations of your chosen assessment of dose. Please make it clear in the methods that dosage was assessed in a very low resolution fashion and provide a short justification for this method of dose assessment.

**Response:** We thank the reviewer for raising this methodological point. We added text to the Discussion acknowledging that dosage data were self-reported, retrospective, and recorded in broad categorical ranges, which may introduce recall inaccuracies (p. 24, L 526-533). In the Methods section, we added a one-sentence justification explaining that simplified dose categories were used to enhance participant comprehension and completion rate, and moved this justification to the 'Survey Instrument' subsection for clarity (p. 5, L152-154).

**Reviewer #3:** Overall Assessment:
This study addresses an important and understudied area in psychedelic research by examining the knowledge, perceptions, and experiences of autistic adults with psychedelics. The work fills a significant gap in our understanding of how this population might respond to and benefit from psychedelic therapies. While the research provides valuable insights into autistic adults' perspectives on psychedelics, several methodological concerns limit the generalizability and reliability of the findings.

Strengths:
The authors have undertaken a valuable investigation into a population that has been historically excluded from clinical trials despite facing high rates of co-occurring mental health conditions. The large sample size of completed surveys provides substantial data on autistic adults' experiences with psychedelics. The co-production approach involving autistic advisors in survey development demonstrates appropriate community engagement and enhances the accessibility of the research instrument. The finding that a majority of participants had used psychedelics, with the majority reporting mental health benefits, provides important preliminary evidence about real-world usage patterns in this population.

**Response:** We sincerely thank the reviewer for the positive evaluation of our manuscript and the detailed, constructive recommendations provided in the attached file. We have addressed these specific points below.

Major Concerns:
**Comment 1:** The recruitment strategy and self-report paradigm likely introduced significant selection bias toward higher-functioning autistic individuals. Online surveys requiring English fluency and the ability to provide detailed self-reports naturally exclude individuals with greater support needs. The predominantly female sample likely interacts with autism support levels in complex ways that remain unaddressed in the analysis. Most critically, the study fails to account for autism support needs levels (formerly functioning levels 1, 2, and 3), which is a significant omission given that individuals requiring substantial or very substantial support may face different risks when using psychedelics without clinical supervision.

**Response:** We thank the reviewer for highlighting this important limitation. We expanded the Discussion to acknowledge potential sampling bias toward higher-functioning autistic adults, given the survey’s online format and English-language requirements Crucially, we now explicitly state that formal autism support needs levels (e.g., DSM-5 Levels 1, 2, or 3) were not assessed, which limits the generalizability of our findings to the broader autistic population (p. 24, L518-525).

**Comment 2:** The retrospective nature of the data collection raises significant concerns about recall bias, as participants may have difficulty accurately remembering experiences that occurred years ago. The study relies entirely on subjective self-report without objective validation or clinical assessment, which limits the reliability of reported mental health improvements and adverse effects.

**Response:** We thank the reviewer for this helpful observation. We have revised the Discussion to more explicitly state that the study relied entirely on retrospective self-reports concerning subjective experiences, dosages, and mental health outcomes, which are subject to recall bias and lack objective clinical validation (p. 24, L526-529).

**Comment 3:** Several survey questions appear potentially leading in their framing toward improvement, such as "Which of these mental health conditions improved after using psychedelics?" and "Did your past psychedelic use help with your mental health concerns?" While this approach is understandable given the study's focus, the limitations of this framing should be more explicitly acknowledged. Also, the temporal direction of expectation effects remains unclear - participants may have had pre-existing expectations about psychedelics that influenced their experiences, or they may have learned about potential benefits after use, which could color their retrospective reporting.

**Response:** We appreciate the reviewer’s comment. We added a note in the Discussion acknowledging that certain items (e.g., improvement-related questions) may have been positively framed, and that participants’ prior expectations about psychedelics could have shaped their responses. We also suggested ways to reduce such effects in future work (p. 24, L529-533).

**Comment 4:** Safety Considerations: While the authors discuss barriers to psychedelic use, they inadequately address the potential risks of unsupervised psychedelic use among autistic individuals, particularly those with greater support needs. The discussion should more explicitly outline safety concerns and emphasize the importance of clinical supervision, especially given the heightened vulnerabilities in this population including sensory sensitivities and co-occurring mental health challenges.

**Response:** We thank the reviewer for emphasizing this crucial point. We expanded the Discussion to address safety concerns related to unsupervised psychedelic use, highlighting potential risks for autistic adults (e.g., sensory sensitivities, co-occurring conditions), which may be amplified in individuals with greater support needs, and the importance of clinical supervision, structured preparation, and integration support in future interventions (p. 25, L534-540).

**Reviewer #4:** This is a well-written manuscript about survey responses related to classical psychedelic and MDMA use for mental health conditions that may co-occur with autism. The survey was administered to individuals who may have identified as being autistic. However, the actual percentage of individuals who endorsed being autistic was not presented in the results or any tables, which is concerning. Aside from this one question asking whether the respondent identified as autistic, there was only one other question related to autism, which was how helpful respondents thought psychedelics and MDMA could be in treating depression, anxiety, or PTSD or other mental health conditions (which aren’t autism), but specifically for people with autism. Regardless if all or none of the respondents actually identified as autistic, in light of the limited scope of the design of the survey, the manuscript fails in three key areas, 1) in terms of its three primary research questions posed at the end of the introduction, 2) in terms of its central aim “to explore knowledge, perceptions, and experiences of autistic adults regarding psychedelics” and 3) in terms of is title, “Knowledge, Perceptions, and Use of Psychedelics among autistic adults: An online survey.” Since it may be too late to develop additional questions that would satisfy these three key areas adequately, the only way forward is to reframe the manuscript and revise the language in these three areas (edit the title, aims, and research questions), to accurately reflect the work that was done so that readers are not disappointed by what they will not find.

**Response:**
We thank the reviewer for this thoughtful and detailed feedback. We revised the title, aims, and research questions to ensure that the manuscript accurately reflects the scope of the study, which focused on co-occurring mental health conditions in autistic adults rather than autism itself. The title now reads: *Knowledge, Perceptions, and Use of Psychedelics for Mental Health among Autistic Adults: An Online Survey.* Corresponding revisions were made in the Introduction and Abstract to clarify that the study examined autistic adults’ perspectives on psychedelics as potential tools for mental health support rather than interventions targeting autism-specific traits. This included refining Research Question 2 to focus specifically on co-occurring mental health symptoms (Title; Abstract, p. 1; p. 4, L107-108)

**Comment 1:** Abstract – Define “neurotypical” at first use, as mental health conditions are forms of neurodivergence and affect neurodevelopmental trajectories, which may confuse readers when insinuating that individuals with depression, etc., are neurotypical.

**Response:**  We defined “neurotypical” at first mention as “typically developing individuals without a diagnosed neurodevelopmental condition” (p. 2, L27-28).

**Comment 2:** Abstract – Be explicit that although the therapeutic potential for treating autism remains under-explored, this work was designed to merely address the co-occurring health conditions in autism and not autism itself.

**Response:** We thank the reviewer for this important comment. We revised the Abstract to explicitly state that the study focused on co-occurring mental health conditions rather than the treatment of autism itself (p. 2, L29).

**Comment 3:** Abstract – The abstract would benefit from a brief description of how “psychedelics” were defined in the survey. Some readers may be curious to know whether MDMA was included, for instance, since there has been some research with MDMA in this population.
**Response:** We thank the reviewer for this suggestion. We added a clarification in the Abstract specifying that “psychedelics” included classical psychedelics such as psilocybin and LSD, as well as MDMA, consistent with the survey definition (p. 2, L32-33).

**Comment 4:** Introduction – This is what I wrote before I discovered the limited scope of the survey: “The first paragraph reads as if the objective of the study is to determine how individuals with autism might merely treat their co-occurring mental health conditions with psychedelics. There is no mention of treating the symptoms of autism itself, how autism is currently treated, gaps in knowledge with treatments, etc., to lead up to potential for psychedelics, including MDMA, as a promising treatment for autism. It isn’t until the final paragraph of the introduction, when the three primary research questions are introduced, does the reader feel oriented, and relieved, that “opinions on using psychedelics as a treatment for their symptoms” indeed falls within the scope of the study objectives.” This original comment shows what readers think the manuscript was aiming to do, when in reality, it was true that the objective was merely to determine how autistic individuals treat co-occurring mental health conditions. Therefore, instead of following my original instructions prior to learning about the limited scope, please be more explicit that the objective is not about autism itself. This will be less problematic after the title, aims, and research questions are revised.
**Response:** We agree with the reviewer that clarification was needed. We revised the Introduction to emphasize that the objective was to explore autistic adults’ perceptions of psychedelics for co-occurring mental health conditions rather than autism-specific symptoms (p. 4, L102-103).

**Comment 5:** Introduction – For greater accuracy, ayahuasca is not a classical psychedelic, but a brew containing a classical psychedelic (DMT). It also should not be capitalized.

**Response:** We thank the reviewer for noting this. We revised the phrasing to “ayahuasca, a brew containing the classical psychedelic DMT,” and removed capitalization throughout (p. 3, L69).

**Comment 6:** Methods – “CAMH” is not defined at first mention but is defined at the end of the methods. Perhaps, as a solution to this small issue, that final section in the methods could be brought up and combined with the Participants section so the reader also has the information related to ethical approvals immediately with the participants, which is more standard.

**Response:** We thank the reviewer for this helpful stylistic suggestion. We defined “Centre for Addiction and Mental Health (CAMH)” at first mention and removed redundant definitions (p. 4, L114). In accordance with the Journal Requirements, the Ethics Statement remains at the beginning of the Methods section. (p. 4, L113-119).

**Comment 7:** Results – A reminder to include the percent of respondents who identified as autistic. Were only the responses from this percent used in the rest of the manuscript? Or were all responses used, regardless if the respondent identified as autistic? If the latter, please restructure findings to focus only on those who responded as identifying as autistic and include not only the number of completers of the survey, but also the number of individuals who identified as autistic as the final number of respondents analyzed.

**Response:** We thank the reviewer for raising this point. We clarified that all participants in the final sample self-identified or were professionally diagnosed as autistic, consistent with inclusion criteria, and reported that 100% of analyzed participants identified as autistic (p. 6, L202-203).

**Comment 8:** Results – This sentence is missing “co-occurring” before mental health conditions: “A significant portion of the sample reported having a history of mental health conditions (96.6%)”
**Response:** We thank the reviewer for catching this detail. We added “co-occurring” before “mental health conditions,” as suggested (p. 6, L210).

**Comment 9:** Results – I’m not sure I understand this sentence, “…who initially endorsed persisting negative effects, four did not describe substantive psychedelic-related issues (e.g., “Good,” “Leading questions,” “Initially depressed but became better,” “Hallucination from 1980s marijuana”).” I think the “e.g.” is meant to be an “i.e.” here, to show what the four descriptions were. I also think these four descriptions could be removed as they seem to do more to confuse than add to the understanding of the sentence. Or just keep “initially depressed but became better” after “e.g.,” with the other three removed. Moreover, it’s also unclear what is meant by “initially endorsed” here. If an earlier item also asked about persisting negative effects for some reason, this should be made explicit.
**Response:** We thank the reviewer for this clarification. We removed the unnecessary examples, retaining only “initially depressed but became better.” We also clarified the phrasing by integrating the explanation directly into the sentence, explaining that “initially endorsed” refers to participants who first indicated persisting negative effects before elaborating in open-ended responses (p. 17, L298-301).

**Comment 10:** Discussion – The discussion must address the key limitation of the survey, that no questions were asked about how psychedelics or MDMA affect autism itself. Moreover, this limitation should reveal that only one question related to autism was posed in the survey and describe what that question asked. These limitations may be offset by what I see as the most powerful and interesting finding in the paper, which was that “The most frequently reported change was improved relationships, as noted by 63.4% of participants,” this is the greatest contribution of the work and especially relevant for autism/ASC.

**Response:** We agree with the reviewer that this limitation should be explicitly addressed. We added a paragraph in the Discussion clarifying that the survey did not include questions about autism-specific traits, as it was designed to examine co-occurring mental health conditions. We also highlighted that the frequent reports of improved relationships (63.4%) may suggest meaningful social benefits deserving of further study (p. 25, 541-547).

**Comment 11:** Discussion – Instead of the word “trip” use “experience”

**Response:** We replaced all instances of “trip(s)” with “experience(s)” throughout the manuscript to maintain a consistent professional tone (p. 22, L437).

**Additional factual and stylistic edits (per co-author Aaron Orsini):**

We have incorporated three minor but important co-author edits for factual precision and stylistic refinement:

1. MDMA pilot study (Introduction, p. 3, L87-88):
   Revised to “N = 12; 8 received MDMA in the randomized, double-blind phase” to more accurately describe the MAPS 2018 crossover design.
2. UCL survey participant count (Introduction, p. 4, L93):
   Corrected “approximately 250 participants” to “233 participants” based on the published UCL 2024 study.
3. Adverse-event phrasing (Introduction, p. 4, L97-98):
   Updated “bad experiences” to “acute adverse events and persisting negative effects” to improve scientific precision and align with standard terminology.

These clarifications were integrated directly into the Introduction section and do not affect the study’s results or interpretations.
